# Flame Retardant Epoxy Composites on the Road of Innovation: An Analysis with Flame Retardancy Index for Future Development

**DOI:** 10.3390/molecules24213964

**Published:** 2019-11-01

**Authors:** Elnaz Movahedifar, Henri Vahabi, Mohammad Reza Saeb, Sabu Thomas

**Affiliations:** 1 Department of Polymer Engineering, Amirkabir University of Technology-Mahshahr Campus, Mahshahr 424, Iran; el.movahedifar@gmail.com; 2 Université de Lorraine, CentraleSupélec, LMOPS, F-57000 Metz, France; 3 Laboratoire Matériaux Optiques, Photoniques et Systèmes, CentraleSupélec, Université Paris-Saclay, 57070 Metz, France; 4 Departments of Resin and Additives, Institute for Color Science and Technology, Tehran P.O. Box 16765-654, Iran; 5 School of Chemical Sciences, MG University, Kottayam, Kerala 686560, India; sabuthomas@mgu.ac.in

**Keywords:** epoxy, Flame Retardancy Index (FRI), fire retardancy, cone calorimetry

## Abstract

Nowadays, epoxy composites are elements of engineering materials and systems. Although they are known as versatile materials, epoxy resins suffer from high flammability. In this sense, flame retardancy analysis has been recognized as an undeniable requirement for developing future generations of epoxy-based systems. A considerable proportion of the literature on epoxy composites has been devoted to the use of phosphorus-based additives. Nevertheless, innovative flame retardants have coincidentally been under investigation to meet market requirements. This review paper attempts to give an overview of the research on flame retardant epoxy composites by classification of literature in terms of phosphorus (P), non-phosphorus (NP), and combinations of P/NP additives. A comprehensive set of data on cone calorimetry measurements applied on P-, NP-, and P/NP-incorporated epoxy systems was collected and treated. The performance of epoxy composites was qualitatively discussed as *Poor*, *Good*, and *Excellent* cases identified and distinguished by the use of the universal Flame Retardancy Index (FRI). Moreover, evaluations were rechecked by considering the UL-94 test data in four groups as V0, V1, V2, and nonrated (NR). The dimensionless FRI allowed for comparison between flame retardancy performances of epoxy composites. The results of this survey can pave the way for future innovations in developing flame-retardant additives for epoxy.

## 1. Introduction

Innovations are mainly born in a very disciplined manner, but sometimes they arise from serendipity. Regardless of the origin of innovative materials and systems, the identification and classification of systems in terms of explanatory variables requires the use of universal, well-accepted criteria. Nowadays, epoxy-based composites are elements of advanced systems [1,2,3]. There has been continued interest in the use of epoxy for developing a wide variety of general- and specific-purpose products such as adhesives, coatings, and medical devices thanks to the versatility of this thermosetting material [4,5,6,7]. Nevertheless, research outcomes reveal that epoxy is highly flammable, and one principally requires flame retardant materials for applications where epoxy should stand against fire [8,9,10,11,12]. In general, it has been understood that careful selection of additives is the first step in development of flame retardant polymer composites, but the performance of the material may additionally depend on the type and the amount of additives used individually or simultaneously [13,14]. Particularly, flame retardant epoxy composites consisting of phosphorus flame-retardant additives were the subject of different reports [15,16]. Moreover, combination of phosphorus and nonphosphorus additives was considered in the quest of higher flame retardancy performance [17,18,19]. In almost all reports, however, there was a lack of a correlation between the crosslinking state of resin in the presence of additives and flame retardancy. 

In a previous work, we used two dimensionless indexes to correlate cure state with corrosion inhibition and flame-retardant properties of epoxy/Fe_3_O_4_ nanocomposites [20]. By the use of dimensionless Cure Index [21] and dimensionless Flame Retardancy Index (FRI) [22], it was demonstrated that the quality of cure in epoxy composites (*Poor*, *Good*, or *Excellent*) can be correlated to the performance of flame retardancy (*Poor*, *Good*, or *Excellent*). The FRI was also powerful in exploring the complementary actions of mineral and organic additives in polymer systems in terms of the peak of HRR (pHRR), the total heat release (THR), and the time to ignition (TTI) of neat polymer and polymer composites [23]. In this work, with the aim of recognizing the future ahead of innovations in flame-retardant epoxy composites, reports on flame-retardant epoxy composites were comprehensively reviewed and then classified as a function of their flame retardancy performance by the use of the FRI criterion. Classification was performed on account of phosphorus (P)-, nonphosphorus (NP)-, and combined P/NP-incorporated epoxy composites. In each class, comprehensive master tables were provided in which the polymer matrix, the additives, the content of additives, and cone calorimetry data including TTI, THR, and pHRR and the calculated FRI values were summarized. Moreover, the available UL-94 test data were provided and plotted similar to the FRI curves, but in four groups of V0, V1, V2, and nonrated (NR).

## 2. Epoxy Resins Containing Phosphorus-Based Flame Retardants

According to the literature, a variety of phosphorus-based flame retardants have been used in epoxy resins. Table 1 summarizes pHRR, THR, and TTI and the FRI values of epoxy/P systems. The percentage of incorporated flame retardant (FR) as well as the results of limiting oxygen index (LOI) and UL-94 test are given.

**Figure 1:**
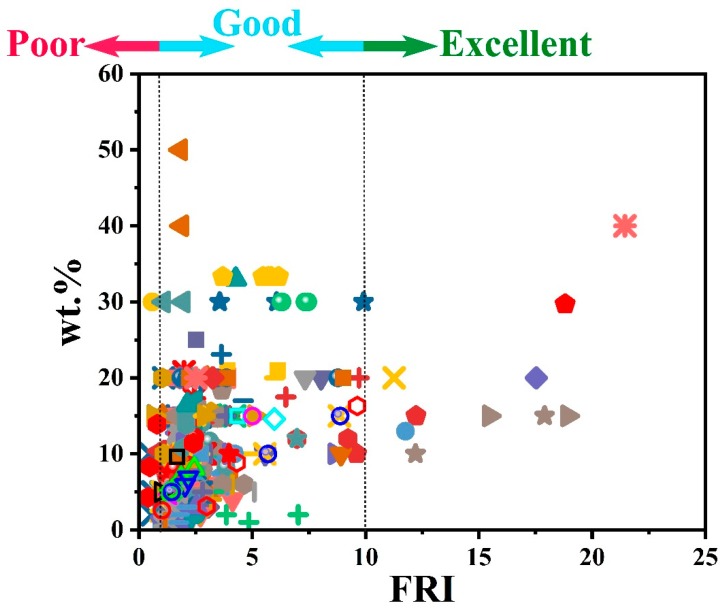
Flame retardancy analysis of epoxy resins containing phosphorus flame retardants in terms of the FRI values as a function of P type and content. Symbols are indicative of different types of phosphorus flame retardant used. Hollow symbols are indicative of fiber-incorporated composites with details earlier given in the bottom of Table 1 as *a* to *l* notes. Here: 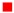 FP1-4, FP1-6, FP1-8 [24], 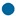 DPO-PHE-11.68, DOPO-PHE-12.03 [25], 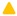 DOPO-T-2.34, DOPO-T-4.67, DOPO-T-6.99, DOPO-T-9.34 [26], 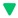 AEPP-5, AEPP-10, AEPP-15 [27], 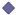 DiDOPO-3 [28], 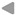 DiDOPO-10, DiDOPO-11 [29], 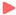 DiDOPO-7 [30], 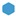 DiDOPO-1, DiDOPO-5, DiDOPO-10 [31], 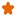 DiDOPO-1, DiDOPO-5, DiDOPO-10, DiDOPO-15, DiDOPO-20 [32], 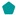 PPMS-15, PPMS-EG-15 [33], 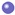 PPMS-MWCNT-5, PPMS-MWCNT-10, PPMS-MWCNT-15, PPMS-15 [34], 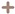 DPIPP-7.5, DPIPP-15, DPPIO-7.5, DPPIO-15 [35], 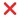 IDOP-5, IDOP-10, IDOP-15 [36], 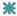 PPAP-5 [37], 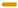 AlPBu-10, AlPBu-11, AlPBu-12 [38], 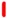 MPL-DOPO-2.5, MPL-DOPO-5, DDM-DOPO-2.5, DDM-DOPO-5 [39], 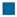 ATZ-6 [40], 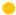 P-KC-30, DOPO-30 [41], 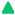 DHPP-OH-BAC-5, DHPP-OH-BAC-10, DHPP-OH-BAC-15 [42], 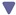 PPAP-5, PPAP-10, PPAP-20 [43], 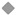 [Dmim]Tos-2.4, [Dmim]Tos-4, [Dmim]Tos-7.5 [44], 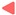 MPhP-10, MPhP-15, MPhP-20 [45], 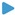 MDOP-0.96, MDOP-1.9, MDOP-3.75, MDOP-7.24 [46], 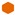 AlPi-7, MPP-7 [47], 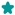 A-BP-9 [48], 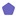 CLEP–DOPO–POSS-2.91 [19], 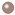 CuPP-1, CuPP-2, CuPP-4, CuPP-6, CuPP-8 [49], 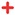 DOP-ABZ-15, DOP-ABZ-17.5, DOP-ABZ-20 [50], 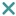 DOPO-7.11, BPD-3.38, BPD-6.71, BPD-10.04, BPD-13.41 [51], 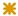 DOPO-7.7, HPCP-8.2 [52], 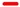 DOPO-TPMP-2.5, DOPO-TPMP-5, DOPO-TPMP-7.5, DOPO-TPMP-10 [53], 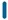 HB-DPPA-2 [54], 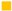 APP-21, EDA-APP-21 [55], 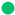 CP-6B-3 [56], 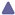 PM-2, PM-6, PM-βCD-2, PM-βCD-6 [57], 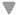 PSA-10, PSA-20 [58], 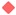 BPA-BPP-9 [59], 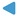 DOPO-9.1, PEPA-9.1, DOPO-PEPA-5.7, DOPO-PEPA-7.4, DOPO-PEPA-9.1 [60], 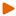 DOPO-POSS-2.5, DOPO-POSS-5, DOPO-POSS-10 [61], 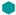 HPCTP-7.46, HPCTP-11.19, HPCTP-14.92, DOPO-6.97, DOPO-10.46, DOPO-13.94 [62], 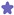 TP-12.42, TNTP-14.36 [63], 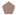 DOPO-7, BNP-7, BNP-11, BNP-14.7, BNP-18.4 [64], 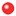 DOPO-7, DTB-7, DTB-10, DTB-15, DTB-20 [65], 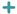 DOPO-7.7, HPCP-8.2 [66], 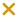 DOPO-7.1 [67], 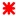 DOPO-7, DOPO-TMT-7, DOPO-TMT-10.4, DOPO-TMT-13.9, DOPO-TMT-17.3, DOPO-TMT-20.8 [68], 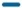 HMCP-3.4, HMCP-6.8, HMCP-10.2, HMCP-13.6, HMCP-17 [69], 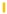 DOPO-bp-3.4, DOPO-bp-6.7, DOPO-bp-13.5 [70], 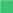 CTP-DOPO-10.6 [71], 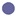 PMTMPS-11 [72], 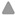 PUTMPS-12, [73], 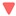 APHP-2, APHP-4, APHP-6 [74], 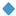 APHP-6, DOPO-6 [75], 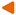 HP-1001-COOH-10, HP-1001-COOH-20, HP-1001-COOH-30, HP-1001-COOH-40, HP-1001-COOH-50 [76], 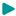 TAD-4 [77], 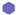 DOPO-10, TAD-6, TAD-8, TAD-10, TAD-12 [78], 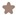 PAz-APP-10, PAz-APP-15 [79], 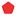 DETA-APP-10, DETA-APP-15 [80], 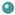 DOPO-8.3, Trif-DOPO-11.7, Trif-DOPO-14 [81], 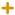 TOD-2, TOD-4, TOD-6 [82], 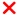 DOPO-DDM-10, DOPO-DDE-10, DOPO-DDS-10 [83], 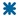 DPP-POSS-5, DPOP-POSS-5, DOPO-POSS-5 [84], 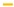 ATH-DOPO-10, ATH-DOPO-20, pATH-DOPO-10 [85], 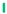 BPS-BPP-9 [86], 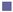 PN-15, PSi-25 [87], 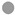 BDMPP-14 [88], 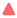 ATCP-15 [89], 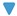 ATCP-15 [90], 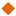 DOPO-4.5, DOPO-ABZ-7.5, DOPO-ABZ-10 [91], 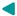 DMT-3.3, DMT-6.6, DMT-10, DMT-13.5, DMT-17 [92], 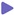 APP-10, APP-MMT-10 [93], 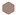 DOPO-6, DOPO-MMT-6 [94], 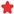 APHP-10, BDP-10 [95], 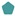 FIPF-20, FTBF-20 [96], 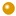 PPDAB-10 [97], 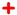 BP-5, BP-9, BP-15 [98], 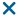 PS-APP-2, PS-APP-5, PS-APP-10, PS-APP-15, PS-APP-20 [99], 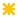 DOPO-POSS-2.5, DOPO-POSS-5, DOPO-POSS-10 [100], 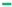 DOPO-POSS-2.5, DOPO-POSS-5, DOPO-POSS-10 [100], 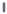 DOPO-POSS-2.5, DOPO-POSS-5, DOPO-POSS-10 [101], 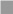 DOPO-5 [102], 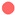 DOPO-6.3 [103], 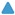 DOPO-6.3 [104], 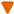 APP-MMT-10 [105], 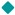 PEPA-5.2, APP-2.9, DOPO-6.3 [106], 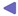 PCPBO-5, PCPBO-10, PCPBO-15, PCPBO-20 [107], 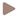 APP-15, GMA-APP-15 [108], 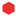 APP-12, MAPP-12 [109], 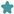 APP-12 [110], 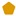 HAP-DOPO-9.3, HAP-DOPO-15.47 [111], 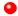 TGIC-DOPO-6.1, TGIC-DOPO-8.1, TGIC-DOPO-10.2, TGIC-DOPO-12.2 [112], 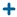 DOPP-19.6, DOPI-23.1 [113], 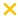 PMPC-10, PMPC-15, PMPC-20 [114], 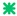 DOPO-5 [115], 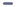 SIEPDP-Mg-Al LDH-4 [116], 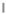 CBz-10, CBz-15, CBz-20 [118], 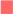 APP-5 [117], 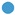 DOPMPA-10, DOPMPA-13 [119], 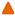 MFR-10, MFR-15, MFR-20 [9], 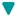 DOPO-COFs-0.4, DOPO-COFs-0.8, DOPO-COFs-1.6, DOPO-COFs-3.2, COFs-3.2 [17], 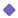 Mel-APP-20 [120], 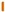 FR-1 [121], 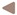 ArPN_2_-15, ArPO_2_-15, ArOPN_2_-15.6, ArOPO_2_-15.6 [122], 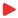 PMAIL-6 [123], 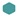 oDOPI-13.81, PZ -10.8, MPP-15 [124], 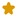 AHP-5 [125], 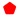 Mel-APP-29.7 [126], 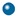 MPAlP-20, MPZnP-20, MPMgP-20, MPP-20, AlPi-Et-20, DOPAc-Bu-20 [127], 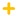 HPCTP-5, HPCTP-10, HPCTP-15 [128], 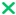 HPCTP-15 [129], 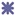 TPP-MMT-5 [130], 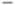 TPP-MMT-5 [130], 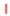 TPP-MMT-5 [130], 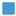 *hb*PPE-10, *hb*PPE-20 [131], 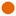 PZS-3, PZS@SrSn(OH)_6_-3 [132], 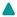 PEPA-TMAC-16.5, PEPA-TMAC-33 [133], 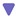 PCPS-1, PCPS-3, PCPS-5 [134], 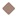 BP1-5, BP2-5, BP3-5, BP4-5, BP5-5 [135], 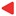 SDPS-10.4 [136], 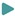 AOPH-NR-4.25, AOPH-C1-4.25, AOPH-C2-4.25, AOPH-C3-4.25 [137], 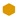 BHAAPE-5, BHAAPE-10, BHAAPE-20 [138], 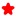 APP-10 [139], 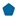 PZS-2, PZS@MoS_2_-2, PZS@MoS_2_-3 [140], 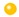 DBPDA-βCD-3 [141], 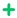 BP-PZN-0.5, BP-PZN-1, BP-PZN-2, BP-Bulk-2 [142], 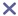 HPPA-2, HPPA-SH-mSiO_2_-2 [143], 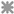 P-MOF-0.5, P-MOF-1, P-MOF-2 [144], 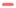 CZrP-2, CZrP-4, CZrP-6, ZrP-6 [145], 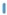 DMMP-HNT-20 [146], 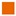 S600-20, AlPi-20, MPP-20 [147], 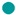 SiO_2_@PZM-1, SiO_2_@PZM@Cu-1, SiO_2_@PZM@Cu-2 [148], 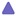 FR@PZS-0.5, FR@PZS-1, FR@PZS-3, PZS-3 [149], 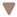 APP-5 [150], 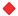 DOPO-POSS [151], 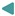 APP-30, M(APP & PER)-30 [152], 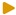 TPPi-15, TPPa-15, TPPO-15 [153], 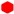 PMP-11.4, DOPO-13.9, RP-4.3, OP-8.3 [154], 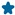 IFR-30, IFR-30, IFR-30 [155], 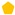 BPE-33.3, EPE-33.3, BBPE-33.3, BOPE-33.3, HBPE-33.3 [156], 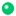 IFR-30, IFR-30, IFR-30, IFR-30 [157], 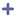 EGM-5, EGM-15 [158], 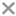 PCTS-Fe-OMMT-1, PCTS-Fe-OMMT-3, PCTS-Fe-OMMT-5 [159], 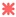 APP-20, APP-40 [160], 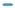 DOPOph-RGNO-1, DOPOph-RGNO-2, DOPOph-RGNO-3 [161], 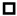 Mel-APP-9.59 [120], 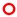 FP1-2.6 [24], 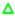 PEC-5.2, PEC-6.9, PEC-8.1 [162], 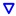 DOPP-5.9, DOPI-6.9 [113], 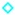 Mel-APP-14.6 [126], 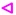 IFR-4.7 [163,164], 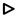 APP-5 [150], 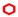 APP-3.15, APP-8.88, APP-16.32 [165], 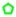 MP-5, DOPO-5 [166], 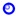 IFR-5, IFR-10, IFR-15 [167], 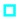 APP-15 [168], 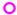 APP-15 [168].

A brief yet informative view of the effect of the used P family of FRs on the flame retardancy performance of epoxy resins is given in Figure 1. It is apparent from the figure that all sorts of behavior, including *Poor*, *Good*, and *Excellent* flame-retardant performance, are achieved. This is the characteristic of dependency of flame retardancy performance on both the type and the content of the P type of FR. It can be observed that the majority of epoxy systems contains less than 20 wt.% of phosphorus flame retardants. For instance, a compromise between FRI and FR loading percentage was achieved by incorporation of encapsulated ammonium polyphosphate (APP-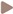) at 15 wt.% with an FRI value of 19. Detailed information about the type of phosphorus flame retardants was provided to the reader in the caption of Figure 1. Thus, innovations in design and manufacture of P type FR for epoxy should carefully meet the requirements based on the lesson learned from the multivariable behavior of flame retardancy brought about by P-type FR additives. Precise detection of the performance of each class of P-type FR in this table from one side and the chemical structure of the used FR from the other side should be balanced towards a high-performance FR for developing flame-retardant epoxy composites.

**Figure 2:**
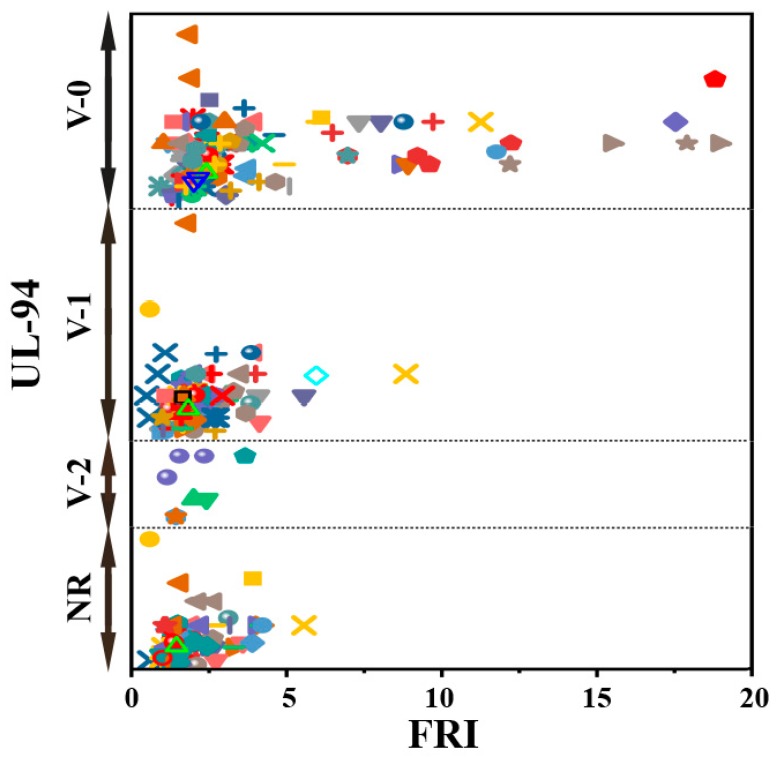
Flame retardancy analysis of epoxy resins containing phosphorus flame retardants in terms of the FRI values as a function of UL-94 test results. Symbols are indicative of different types of phosphorus flame retardant used. Hollow symbols are indicative of fiber-incorporated composites with details earlier given in the bottom of Table 1 as *a* to *l* notes. The vertical variation in each category, i.e., V-0, V-1, V-2, and NR, is schematically representative of the amount of additive used. For example, among two data distinguished by different symbols having the same or very close FRI values (horizontal quantity) in a given category (e.g., V-1), which have different vertical quantity both revealed V-1 behavior in UL-94 test, but the upper was an FR used in more quantity in preparation of epoxy composites.

Although variation of FRI values according to the composition reflects the flame retardancy of epoxy composites from cone calorimetry angle (the most reliable test among those normally used for analysis of performance of flame retardants), other types of flame tests would give more insights into the real effect of one or complementary actions of two or more P type FR additives in epoxy. Based on available data, a brief view of the effect of the used P-based FRs on the flame retardancy performance of epoxy resins as a function of UL94 results is given in Figure 2. The distribution of data in this figure gives useful information about the efficiency of the FR system in harsh conditions. For instance, this figure suggests that V-0 performance in UL94 can be achieved even at the *Poor* category of flame retardancy performance in terms of FRI. It appears that it is not possible to roughly correlate the obtained results in UL94 to those obtained in cone calorimetry tests.

**Figure 3:**
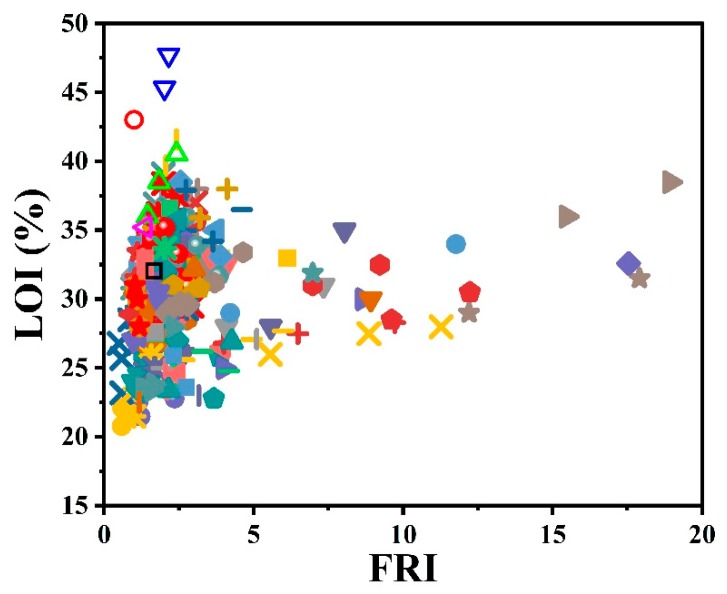
Flame retardancy analysis of epoxy resins containing phosphorus flame retardants in terms of the FRI values as a function of LOI test results. Symbols are indicative of different types of phosphorus flame retardant used. Hollow symbols are indicative of fiber-incorporated composites with details earlier given in the bottom of Table 1 as *a* to *l* notes.

Another test of importance is the limiting oxygen index (LOI), which is demonstrative of flammability. A self-extinguishing behavior is expected when the LOI value is higher than 28. A brief overview of the effect of the used phosphorus-type flame retardants on the flame retardancy performance of epoxy resins as a function of LOI results is given in Figure 3. Surprisingly, the highest value obtained in LOI testing is located in the *Good* zone of FRI. The collection of data with FRI values below 5, where LOI% varies depending on the type of phosphorus additive and undoubtedly the content, is hidden behind these symbols.

## 3. Epoxy Resins Containing Nonphosphorus Flame Retardants

According to the literature, a variety of nonphosphorus FRs have been used in epoxy resins. Table 2 summarizes pHRR, THR, and TTI and the FRI values of epoxy/NP systems. The percentage of incorporated FR as well as the results of LOI and UL-94 test are also given for comprehensive determination of the behavior of this family of epoxy composites.

**Figure 4:**
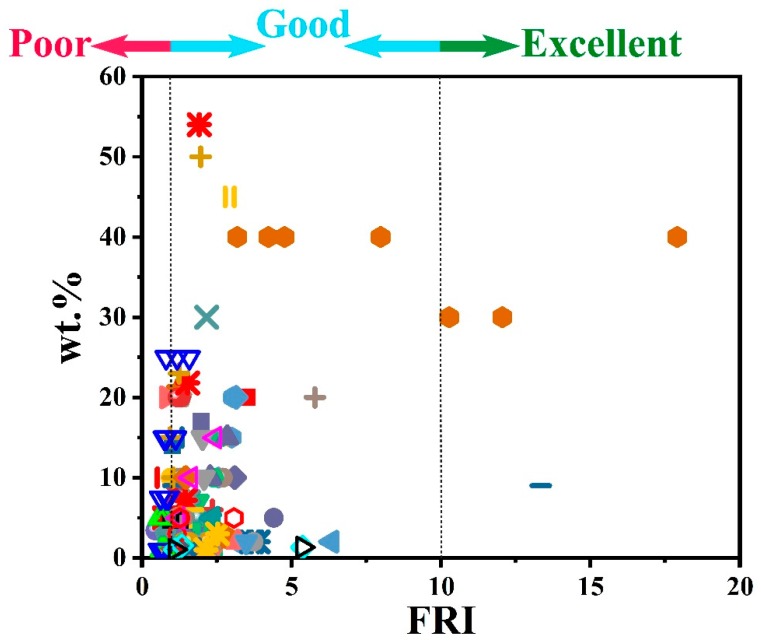
Flame retardancy analysis of epoxy resins containing nonphosphorus flame retardants in terms of the FRI values as a function of NP type and content. Symbols are indicative of different types of NP type of FR used. Hollow symbols are indicative of fiber-incorporated composites with details earlier given in the bottom of Table 1 as notes *a* to *h*. Here: 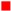 3TT-3BA-20 [169], 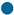 GN-3 [28], 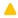 MWCNT-0.8 [29], 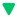 OMMT-7 [30], 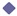 OLDH-1, OLDH-5, OLDH-10 [31], 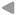 MgAl-LDH-2, ZIF8-2, ZIF8@MgAl-LDH-2, ZIF67-2, ZIF67@MgAl-LDH-2 [170], 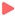 TAT-20 [52], 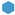 TNB-1, TNB-5, TNB-10, TNB-15, TNB-20 [171], 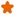 Cu_2_O-21 [55], 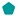 MH-3, [56], 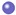 TN-3.42 [63], 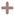 EG-20 [66], 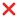 TMT-8 [67], 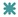 TMT-7 [68], 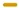 OMMT-1 [77], 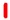 TAIC-10 [78], 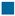 TPT-14 [81], 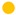 HNT-5, HNT-10, HNT@PDA-5, HNT@PDA-10, HNT@PDA@Fe(OH)_3_-5, HNT@PDA@Fe(OH)_3_-10 [172], 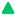 MMT-6 [94], 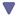 OPS-5 [102], 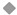 OPS-4.1, PPSQ-4.1 [103], 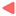 OPS-4.1, OAPS-4.6 [104], 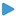 OPS-4.1 [106], 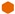 ATH-40, C-40, U-40, BA-40, BO-40, MB-30, GB-30 [173], 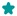 ODPSS-5 [115], 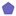 Mg-Al LDH-4 [116], 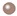 T8POSS-10, TGIC-10 [174], 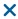 RGO-1 [121], 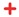 HNT-2, LDH-2, LDH-4, LDH-6 [120], 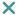 AlO(OH)-30 [124], 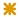 ACS-2, ACS@SnO_2_-2, ACS@SnO_2_@NiO-2 [125], 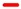 ACS@SnO_2_@NiO-5 [125], 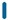 OGPOSS-15 [129], 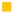 EG-15 [33], 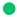 CP-10, CP-15 [130], 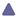 CP-10, CP-15 [130], 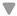 CP-10, CP-15 [130], 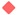 SrSn(OH)_6_-3 [132], 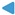 SiO_2_-2, ZIF8-2, ZIF8@SiO_2_-2 [175], 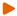 MoS_2_-2, TNT-2, MoS_2_-TNT-1, MoS_2_-TNT-2, MoS_2_-TNT-3 [176], 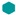 Sep-2, Sep-4, Fe_3_o_4_–Sep-2, Fe_3_o_4_–Sep-4 [177], 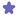 GNO-1, GNO-3, GN-Cu-1, GN-Cu-3 [178], 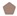 AlO(OH)-20 [147], 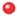 AlO(OH)-20, SiO_2_-20 [127], 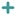 α-MnO_2_-0.5, α-MnO_2_-1, α-MnO_2_-2, δ-MnO_2_-0.5, δ-MnO_2_-1, δ-MnO_2_-2 [179], 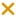 MoS_2_-2 [140], 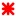 AI-POSS-7.2, AI-POSS-21.8, AI-POSS-54 [180], 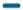 EG-9, HNT-9 [181], 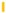 BN 2 μm-45, BT 2 μm-45 [182], 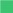 MnO_2_-2, MnO_2_@ZHS-0.5, MnO_2_@ZHS-1, MnO_2_@ZHS-2 [183], 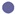 ILFR-5, BN-5, ILFR-fBN-5 [184], 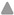 SH-mSiO_2_-2 [143], 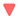 SCF-0.5, SCF-0.7, SCF-1, SCF-1.5 [185], 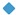 HNT-20 [146], 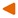 m-Clay-2.5, d-Clay-2.5 [186], 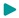 LDH-3, β-FeOOH-3, LDH-β-FeOOH-3 [187], 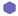 AHTSS-0.5, AHTSS-2, UMTHS-0.5, UMTHS-2 [188], 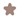 CS-MoS_2_-0.5, CS-MoS_2_-1, CS-MoS_2_-2, MoS_2_-2 [189], 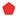 SiO_2_-1 [148], 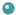 CNT-1, CCNT-1, TCNT-1, LDH-5, OLDH-5, MMT-5, OMMT-5, ATH-5 [150], 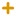 EG-5, EG-10, EG-15, EG-23, EG-50 [190], 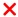 BT-3, BT-5, BFTDA-BT-3, BFTDA-BT-5, APUA-BT-3, APUA-BT-5 [191,192], 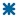 GN-2, Ni–Fe LDH-2 [193], 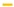 OAPOSS-MMT-2, OAPOSS-MMT-4, OAPOSS-MMT-6 [194], 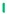 Na-magadiite-3, S-Na-magadiite-3, S-H-magadiite-3, OM-magadiite-3, S-OM-magadiite-3 [195], 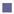 TBBA-17 [154], 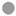 GN-2, Ce–MnO_2_-2, Ce–MnO_2_–GN-2 [196], 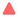 m-SiO_2_-2, Co−Al LDH-2, m-SiO_2_@Co−Al LDH-2 [197], 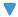 ZnS-2, GN-2, ZnS-GN-2 [198], 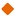 sep idra-2, sep idra-5, sep idra-10, sep anidra-2, sep anidra-5, sep anidra-10 [199], 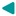 EG-5 [158], 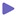 CTS-Fe-OMMT-3, CTAB-Fe-OMMT-3 [159], 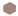 A-MWCNT(Polish)-0.05, A-MWCNT(Polish)-0.1, A-MWCNT(Polish)-0.5, A-MWCNT(Polish)-1, A-MWCNT(Polish)-5, C-MWCNT(Polish)-0.05, C-MWCNT(Polish)-0.1, C-MWCNT(Polish)-0.5, C-MWCNT(Polish)-1, C-MWCNT(Belgian)-0.05, C-MWCNT(Belgian)-0.5, CA-MWCNT(Polish)-0.05, CA-MWCNT(Polish)-0.1, CA-MWCNT(Polish)-0.5, CA-MWCNT(Polish)-1, CA-MWCNT(Polish)-5, A-MWCNT(Belgian)-0.05, A-MWCNT(Belgian)-0.1, A-MWCNT(Belgian)-0.5 [200], 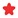 GNO-1 [201], 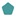 I.30E-3 [160], 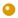 MoS_2_-2,GN-2, MoS_2_-GN-2 [202], 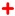 GNO-1 [161], 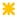 BNO-1, BNO-3 [203], 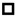 Vis-4.7, Ky-4.7 [163,164], 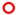 clay-1, clay-3, clay-5 [204], 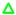 LDH-5, OLDH-1, CNT-1, CCNT-1, TCNT-1, ATH-5 [150], 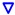 Mg(OH)_2_-1, Mg(OH)_2_-7.5, Mg(OH)_2_-15, Mg(OH)_2_-25, Al(OH)_3_-1, Al(OH)_3_-7.5, Al(OH)_3_-15, Al(OH)_3_-25, ZB-1, ZB-7.5, ZB-15, ZB-25 [205], 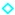 SWCNT-BP-1.06, MWCNT-BP-1.34, CNF-1.57 [206], 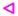 Vis-5, Vis-10, Vis-15 [167], 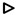 SWCNT-BP-1.06, MWCNT-BP-1.34 [207], 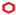 T8POSS-5, TGIC-5 [174].

From the comparison between Table 1 and Table 2, one can simply infer that the NP family is less effective in terms of the flame retardancy of the composite epoxy with respect to the P family of FR. The effect of the used NP-type FR on the flame retardancy performance of epoxy resins can be visually assessed in Figure 4. Moreover, detailed information about the type of NP additives is provided to the reader in the caption of Figure 4. The quality of epoxy composites containing NP additives suggests that even at high loading levels it is difficult to attain very high efficiencies. As an informative case, alumina Trihydrate (ATH, 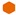) has been used in a wide range of content in development of flame-retardant epoxy nanocomposites. It can be seen that at high loading rate (up to 30 wt.%), it gives the best results, *Excellent* in terms of FRI. It can be concluded that the NP class of additives are not individually responsible for high fire resistance of epoxy.

**Figure 5:**
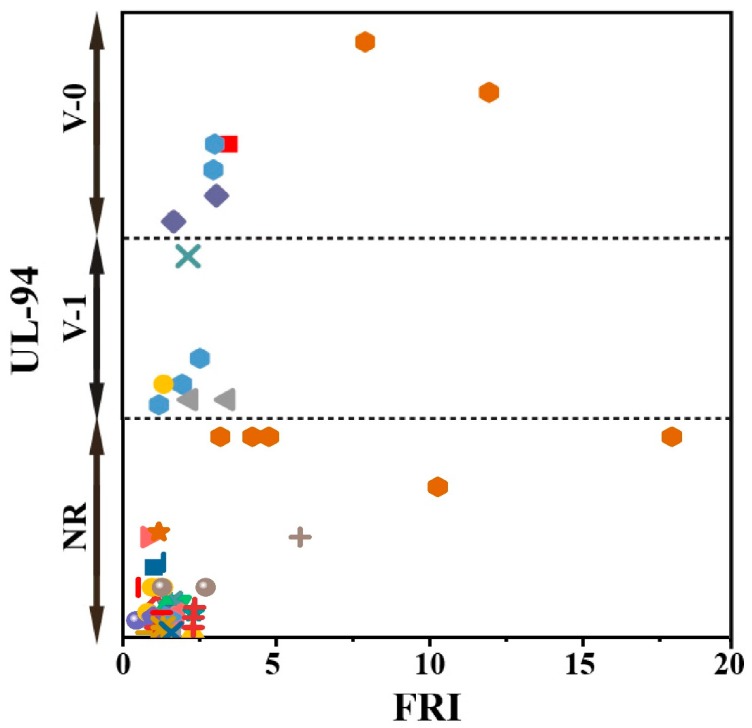
Flame retardancy analysis of epoxy resins containing nonphosphorus flame retardants in terms of the FRI values as a function of UL-94 test results. Symbols are indicative of different types of NP type of FR used in this figure. Hollow symbols are indicative of fiber-incorporated composites with details given in the bottom of Table 2 as notes *a* to *h*. The vertical variation in each category, i.e., V-0, V-1, V-2, and NR, is schematically representative of the amount of additive used. For example, among two data distinguished by different symbols having the same or very close FRI values (horizontal quantity) in a given category (e.g., V-1), which have different vertical quantity both revealed V-1 behavior in UL-94 test, but the upper was an FR used in greater quantity in preparation of epoxy composites.

A brief overview of the effect of the NP used as FR in epoxy composite preparation and on the flame retardancy performance of epoxy resins as a function of UL-94 results is given in Figure 5. Since data are limited and spread over the plot, there is no conclusion about the relationship between FRI (cone calorimetry) and UL-94 analysis to be highlighted. Nevertheless, all sorts of behavior can be seen in the plot, depending on the type and content of NP type of FRs. It is worthy of note that the NR category of UL-94 constitutes a high proportion of the results.

**Figure 6:**
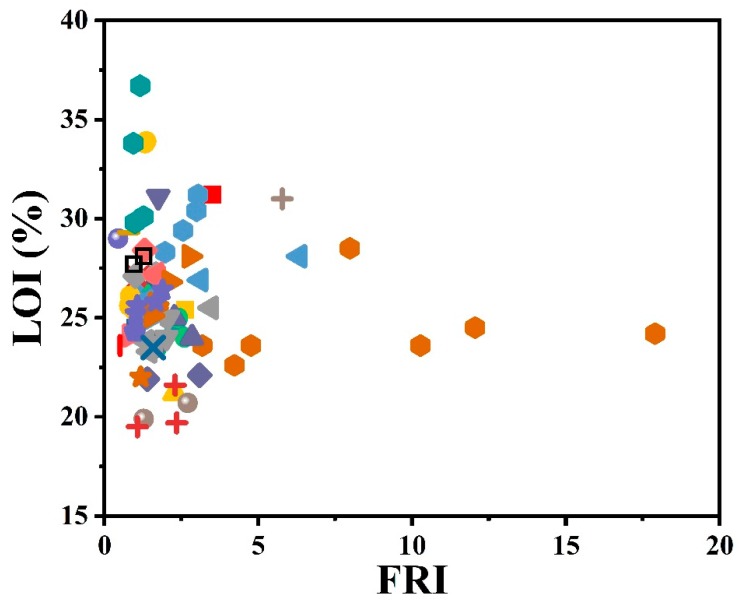
Flame retardancy analysis of epoxy resins containing nonphosphorus flame retardants in terms of the FRI values as a function of LOI test results. Symbols are indicative of different types of NP flame retardant used. Hollow symbols are indicative of fiber-incorporated composites with details given in the bottom of Table 2 as notes *a* to *h*.

A brief overview of the effect of NP-type FR on the flame retardancy performance of epoxy resins as a function of LOI results is given in Figure 6. Surprisingly, the highest value obtained in LOI testing is located in *Poor* zone of FRI. On the other hand, *Excellent* flame retardancy seen at high FRI values has LOI of about 22%. From this perspective, it can be concluded that cone calorimetry is not monotonically representative of the character of FR when used in epoxy.

## 4. Epoxy Resins Containing Combinatorial Flame Retardant Systems

Assessing the flame retardancy performance of P- and NP-incorporated epoxy systems unraveled the inadequacy of using one FR additive alone when a high performance is required. The antagonism or synergism may be the result of using two or more FR systems in a given polymer matrix. In the case of epoxy, there have been some attempts towards combinatorial use of P and NP additives for the sake of higher performance. Table 3 summarizes pHRR, THR, TTI, and FRI values of epoxy/P/NP combinatorial flame-retardant systems. The percentage of incorporated FR as well as the results of LOI and UL-94 tests are also given.

**Figure 7:**
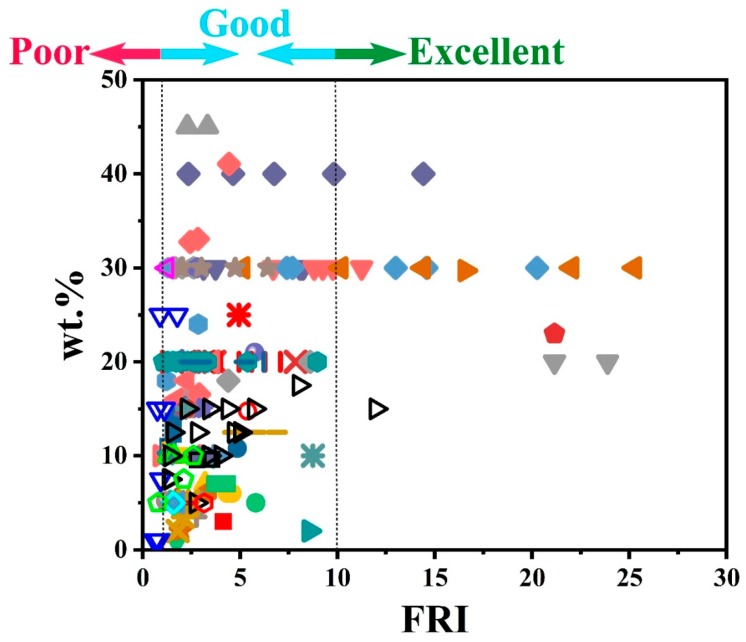
Flame retardancy analysis of epoxy resins containing combinatorial flame retardant systems in terms of the FRI values as a function of combinatorial flame retardants systems retardant type and content. Symbols are indicative of different types of combinatorial flame retardants systems used. Hollow symbols are indicative of fiber-incorporated composites with details earlier given in the bottom of Table 1 as notes *a* to *i*. Here: 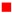 DiDOPO-1.5/GN-1.5 [28], 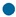 DiDOPO-10/MWCNT-0.8 [29], 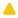 DiDOPO-3.5/OMMT-3.5 [30], 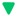 DiDOPO-0.5/OLDH-0.5, DiDOPO-2.5/OLDH-2.5, DiDOPO-5/OLDH-5 [31], 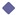 IFR-40, IFR-39/CES-1, IFR-38/CES-2, IFR-37/CES-3, IFR-35/CES-5 [208], 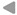 DOPO-15/P-KC-15, DOPO-20/P-KC-10, DOPO-25/P-KC-5 [41], 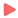 mAPP-5/PER-5, mAPP-5/RCC-5, mAPP-5/ORCC-5 [209], 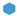 PEPA–TMA-12/MCA-6, PEPA–TMA-16/MCA-8, PEPA–TMA-20/MCA-10 [210], 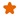 ZIF8-1/MgAl-LDH-1, ZIF67-1/MgAl-LDH-1 [170], 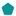 TAT-18/DOPO-2, TAT-16/DOPO-4, TAT-14/DOPO-6, TAT-12/DOPO-8, TAT-18/HPCP-2, TAT-16/HPCP-4, TAT-14/HPCP-6, TAT-12/HPCP-8 [52], 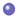 EDA-APP-19/Cu_2_O-2 [55], 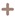 CP-6B-3/MH-0.5 [56], 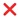 IFR-20, IFR-19.5/HGM-0.5, IFR-19/HGM-1, IFR-18/HGM-2, IFR-16/HGM-4 [211], 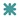 APP-5/PSA-5 [58], 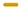 MFAPP-6.25/PER-6.25, MFAPP-6.25/ST-6.25, MFAPP-6.25/OST-6.25 [212], 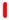 EG-16/DOPO-4, EG-14/DOPO-6, EG-12/DOPO-8, EG-10/DOPO-10, EG-16/HPCP-4, EG-14/HPCP-6, EG-12/HPCP-8, EG-10/HPCP-10 [66], 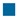 TMT-8.3/DOPO-2.7, TMT-8.2/DOPO-4.1, TMT-8.1/DOPO-5.6, TMT-8/DOPO-7 [67], 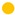 DOPO-3/APHP-3, DOPO-4/APHP-2 [75], 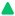 TAD-4/OMMT-1 [77], 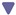 FR-20/APP-10, FR-15/APP-15, FR-12/APP-18, FR-10/APP-20 [213], 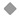 ATCP-15/FRHA-1, ATCP-15/FRHA-3, ATCP-15/FRHA-5 [89], 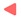 ATCP-15/FRHA-1, ATCP-15/FRHA-3, ATCP-15/FRHA-5 [90], 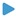 APP-4/MMT-6 [93], 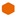 DOPO-5/MMT-1 [94], 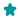 BDP-6.7/PHP-3.3 [95], 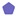 OPS-2.5/DOPO-2.5 [102], 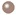 DOPO-3.1/OPS-2.1, DOPO-3.1/PPSQ-2.1 [103], 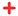 DOPO-3.1/OPS-2.1, DOPO-3.1/OAPS-2.3 [104], 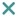 OPS-2.5/DOPO-2.5 [105], 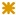 OPS-2.1/PEPA-2.6, OPS-2.1/APP-1.4, OPS-2.1/DOPO-3.1 [106], 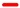 ODPSS-2.5/DOPO-2.5 [115], 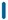 BBO-10/PPA-10 [214], 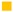 T8POSS-5/TGIC-5 [174], 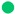 APP-4.83/CoSA-0.17 [117], 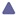 CBz-8/BGN-2, CBz-13/BGN-2, CBz-18/BGN-2 [118], 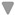 Mel-APP-18/LDH-2, Mel-APP-18/HNT-2 [120], 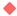 oDOPI-17.76/MPP-15, AlO(OH)-30/oDOPI-11.05, MPP-15/PZ-1.54, AlO(OH)-30/PZ-3.08 [124], 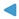 AHP-4.5/ACS@SnO_2_@NiO-0.5 [125], 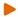 Mel-APP-19.97/Talc-9.73 [126], 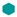 MPP-10/MPZnP-10, AlPi-Et-10/MPZnP-10, DOPAc-Bu-10/MPZnP-10, AlO(OH)-10/MPZnP-10, MPZnP-10/SiO_2_-10, MPP-13.4/MPZnP-6.6, AlPi-Et-13.4/MPZnP-6.6, DOPAc-Bu-13.4/MPZnP-6.6, AlO(OH)-13.4/MPZnP-6.6, SiO_2_-13.4/MPZnP-6.6 [127], 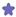 HPCTP-10/OGPOSS-5, HPCTP-7.5/OGPOSS-7.5, HPCTP-5/OGPOSS-10 [129], 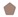 CP-10/TPP-MMT-5 [130], 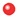 CP-10/TPP-MMT-5 [130], 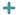 CP-10/TPP-MMT-5 [130], 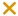 MoS_2_-1/TNT-1 [176], 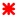 APP-15/PER-HNT-10 [215], 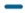 S600-10/AlPi-10, S600-10/AlO(OH)-10, S600-10/MPP-10 [147], 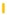 SDPS-5.2/SPDM-5.2 [136], 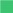 AlPi-4.7/MPP-2.3, AlPi-4.5/MPP-2.25/Al_2_O_3_-0.25 [47], 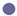 APP-8/CSA-2, APP-7.5/CSA-2.5, APP-6.7/CSA-3.3 [139], 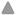 BN 12 μm-33.75/BN 2 μm-11.25, BN 12 μm-33.75/BT 2 μm-11.25 [182], 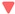 IFR-30, IFR-29.5/FeP-0.5, IFR-29/FeP-1, IFR-28/FeP-2, IFR-27/FeP-3 [216], 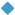 IFR-30, IFR-29.5/αFeOOH-0.5, IFR-29/αFeOOH-1, IFR-28/αFeOOH-2, IFR-27/αFeOOH-3 [217], 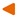 IFR-30, IFR-29.5/iron oxide brown-0.5, IFR-29/iron oxide brown-1, IFR-28/iron oxide brown-2, IFR-27/iron oxide brown-3 [218], 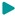 Ni–Fe LDH-2/GN-2 [193], 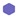 APP-22.5/PER-7.5 [152], 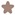 IFR-30, IFR29.5/Fe-OMMT-0.5, IFR-29/Fe-OMMT-1, IFR-28/Fe-OMMT-2, IFR-27/Fe-OMMT-3 [219], 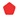 APP-20/I.30E-3 [160], 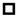 Mel-APP-8.59/LDH-0.96, Mel-APP-8.65/HNT-0.96 [120], 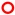 Mel-APP-9.93/Talc-4.84 [126], 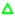, IFR-5/Vis-5, Ky-5/IFR-5 [163,164], 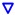 ZB-0.5/Mg(OH)_2_-0.5, ZB-3.75/Mg(OH)_2_-3.75, ZB-7.5/Mg(OH)_2_-7.5, ZB-12.5/Mg(OH)_2_-12.5, ZB-0.5/Al(OH)_3_-0.5, ZB-3.75/Al(OH)_3_-3.75, ZB-7.5/Al(OH)_3_-7.5, ZB-12.5/Al(OH)_3_-12.5 [205], 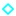 MP-4.5/GN-0.5, DOPO-4.5/GN-0.5 [166], 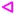 PFR-25/ZB-5 [220], 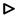 IFR-2.5/Vis-2.5, IFR-2.5/Vis-5, IFR-2.5/Vis-7.5, IFR-2.5/Vis-10, IFR-2.5/Vis-12.5, IFR-5/Vis-2.5, IFR-5/Vis-5, IFR-5/Vis-7.5, IFR-5/Vis-10, IFR-7.5/Vis-2.5, IFR-7.5/Vis-5, IFR-7.5/Vis-7.5, IFR-10/Vis-2.5, IFR-10/Vis-5, IFR-12.5/Vis-2.5, IFR-15/Vis-2.5 [167], 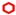 T8POSS-2.5/TGIC-2.5 [174], 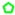 IFR-2.5/Vis-2.5, IFR-3.75/Vis-3.75, IFR-7.5/Vis-2.5 [221].

To give a more meaningful overview of the effect of combined P and NP additives on flame retardancy performance of epoxy, FRI values are calculated by using calorimetric data given in Table 3 and plotted in Figure 7. In this figure, the vertical axis shows the amount of additive system used in preparation of epoxy composites. The plot also reveals that three types of flame retardancy performances are observed, depending on the type of combinatorial systems as well as the amount of FR additives used. Attention should be paid to the fact that even at lower loading levels, careful coupling of one or more P and NP additives could lead to superiority of the FR system used, and there was a possibility for attaining higher performances compared to highly-filled systems (FR content ≥ 40). Thus, careful selection of complementary additives with disciplined loading can result in high flame retardancy performance.

**Figure 8:**
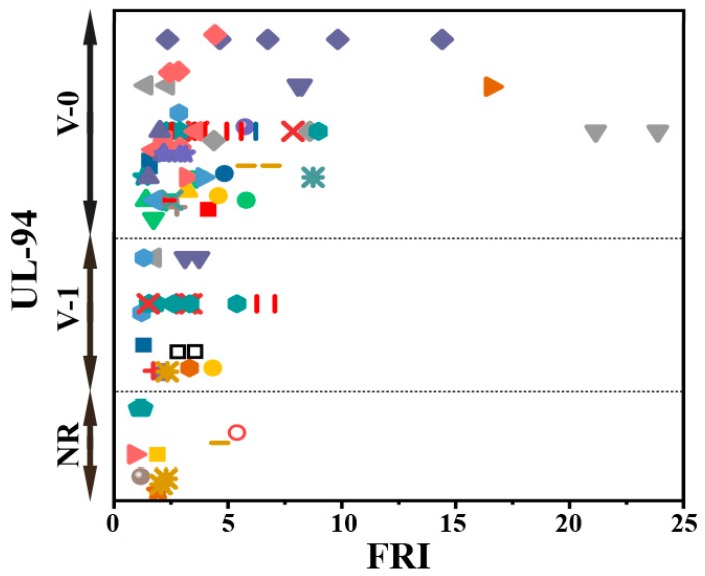
Flame retardancy analysis of epoxy resins containing combinatorial flame retardants in terms of the FRI values as a function of UL-94 test results. Symbols are indicative of different types of combinatorial flame retardants used. Hollow symbols are indicative of fiber-incorporated composites with details given in the bottom of Table 1 as *a* to *i* notes. The vertical variation in each category, i.e., V-0, V-1, and NR, is schematically representative of the amount of additive used. For example, two data distinguished by different symbols have the same or very close FRI values (horizontal quantity) in a given category (e.g., V-1), but higher V-1 behavior in UL-94 testing means the FR was used in greater quantity.

When looking at the UL-94 test results (considering the fact that there were some data in Table 3 for some systems to be plotted and discussed in Figure 8), it can be seen that, except for some data, the whole systems take *Poor* and *Good* labels based on FRI values. It is also interesting to note that for a given category, e.g., V-0, the amount of additive changes the FRI, and UL-94 testing does not make sense of such variations.

**Figure 9:**
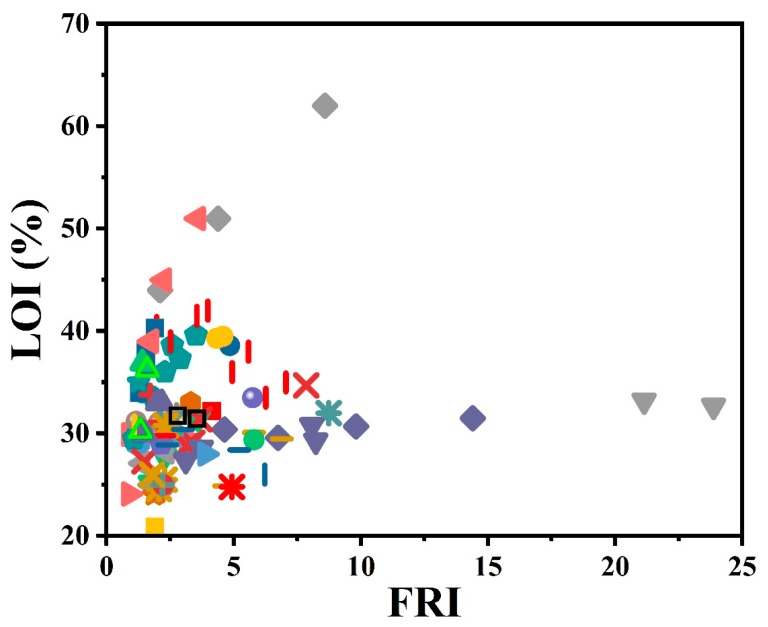
Flame retardancy analysis of epoxy resins containing combinatorial flame-retardant systems in terms of the FRI values as a function of LOI test results. Symbols are indicative of different types of combinatorial flame-retardant systems used. Hollow symbols are indicative of fiber-incorporated composites with details given in the bottom of Table 1 as notes *a* to *i*.

The more interesting outcome of this work is that LOI percent similarly detects *Poor* and *Good* behaviors, not principally *Excellent* performance (Figure 9). This suggests that development of innovative FR additives by combination of P and NP and using highly efficient synthesis routes is the essential step to be taken in the near future for developing flame retardant epoxy composites.

## 5. Concluding Remarks and Future Perspective

**Figure 10:**
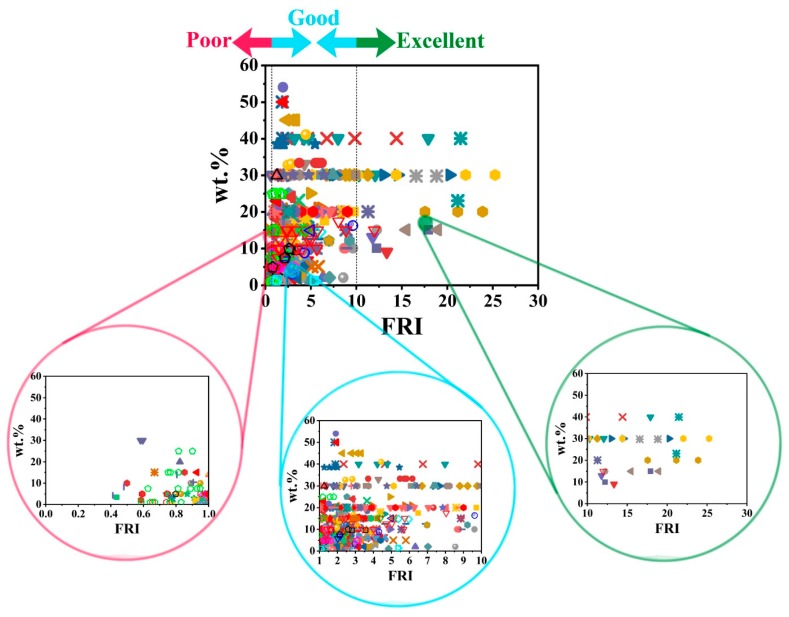
Overall flame retardancy behavior of epoxy resins regardless of the type of flame retardant. *Poor*, *Good*, and *Excellent* efficiencies are magnified to give a close-up of the data distribution.

In previous sections, we categorized the flame-retardant properties of epoxy resins in terms of the universal FRI criterion and the content of flame retardants of three families. We also attempted to find possible correlations between cone calorimetry (reflected in FRI variations), UL-94, and LOI analyses. Since cone calorimetry is the best way to simulate real state combustion of polymers, here, we give a general picture of flame retardancy of epoxy resins (Figure 10). The *Poor*, *Good*, or *Excellent* flame retardancy cases are the result of the P, NP, or P/NP types of flame retardants used in preparation of epoxy composites as well as the FR loading. Each kind of behavior can be visualized by providing a full snapshot of the *Poor*, *Good*, and *Excellent* regions of the FRI to see how closely the data are collected in each zone. Overall, it can be seen that *Poor* and *Good* are the cases for majority of data, while the *Excellent* zone contains limited data. This highlights the difficulty of achieving high flame-retardant efficiency in epoxy composites when merely using flame retardants. Thus, development of innovative flame retardants through blending different FR families and making them reactive towards epoxy may result in a fully cured 3D network with high flame resistance. This requires the knowledge and experience of chemists and engineers who can adjust the performance of the system in a very disciplined manner. Moreover, using bio-based epoxy resins with limited environmental threats would be another solution to the question of “which FR additive(s) meet the requirements of highly flame-retardant epoxy composites?”.

## Figures and Tables

**Table 1:** The flame retardancy performance of epoxy containing phosphorus-based (P) flame retardants in terms of FRI (* the name and percentage of incorporated flame retardant is given after each epoxy resin). Notes *a* to *l* on the bottom of the table are representative of composite systems containing woven or nonwoven fibers.

Epoxy Resins and Incorporated Phosphorus FR *	wt.%	TTI (s)	pHRR (kW·m^−^^2^)	THR (MJ·m^−^^2^)	FRI	LOI	UL94	Ref.
	0	49	1477	118	—	27	NR	[24]
N, N′-diallyl-p-phenylphosphonicdiamide (FP1)	4	46	831	106	1.85	33	NR	[24]
N, N′-diallyl-p-phenylphosphonicdiamide (FP1)	6	42	500	115	2.59	36	V-1	[24]
N, N′-diallyl-p-phenylphosphonicdiamide (FP1)	8	40	587	109	2.22	38	V-0	[24]
	0	31	1068	76	—	23.7	NR	[25]
(bis(4- hydroxyphenyl) methyl) diphenylphosphine oxide (DPO-PHE)	11.68	41	657	59	2.76	32.1	V-0	[25]
1-(bis(4-hydroxyphenyl)methyl)-9,10-dihydro-9- oxa-10-phosphaphenan-threne-10-oxide (DOPO-PHE)	12.03	39	956	57	1.87	30.5	V-0	[25]
	0	47	1208	80	—	22.5	NR	[26]
Reaction between 9,10-dihydro-9-oxa-10-phosphaphenanthrene-10-oxide & cyanuric chloride (DOPO-T)	2.34	38	836	69	1.35	32.5	NR	[26]
reaction between 9,10-dihydro-9-oxa-10-phosphaphenanthrene-10-oxide & cyanuric chloride (DOPO-T)	4.67	36	727	62	1.64	34.6	V-1	[26]
reaction between 9,10-dihydro-9-oxa-10-phosphaphenanthrene-10-oxide & cyanuric chloride (DOPO-T)	6.99	32	629	56	1.86	36.2	V-1	[26]
Reaction between 9,10-dihydro-9-oxa-10-phosphaphenanthrene-10-oxide & cyanuric chloride (DOPO-T)	9.34	30	613	54	1.86	33.4	V-0	[26]
	0	131	495	179	—	21.3	V-2	[27]
Aluminum ethylphenylphosphinate (AEPP)	5	119	254	131	2.41	23.3	V-2	[27]
aluminum ethylphenylphosphinate (AEPP)	10	105	241	124	2.37	25.7	V-1	[27]
aluminum ethylphenylphosphinate (AEPP)	15	91	223	119	2.31	28.2	V-0	[27]
	0	32	827	116	—	21.8	NR	[28]
phenethyl-bridged 9,10-dihydro-9-oxa-10-phosphaphenanthrene-10-oxide derivative (DiDOPO)	3	41	387	104	3.05	32.7	V-0	[28]
	0	32	781	107	—	21.8	NR	[29]
Phenethyl-bridged 9,10-dihydro-9-oxa-10-phosphaphenanthrene-10-oxide derivative (DiDOPO)	10	38	508	83	2.35	38	V-0	[29]
phenethyl-bridged 9,10-dihydro-9-oxa-10-phosphaphenanthrene-10-oxide derivative (DiDOPO)	11	43	441	96	2.65	37.4	V-0	[29]
	0	32	781	107	—	21.8	NR	[30]
phenethyl-bridged 9,10-dihydro-9-oxa-10-phosphaphenanthrene-10-oxide derivative (DiDOPO)	7	36	491	80	2.39	35.7	V-0	[30]
	0	32	781	107	—	21.8	NR	[31]
phenethyl-bridged 9,10-dihydro-9-oxa-10-phosphaphenanthrene-10-oxide derivative (DiDOPO)	1	33	516	116	1.43	24.1	V-2	[31]
phenethyl-bridged 9,10-dihydro-9-oxa-10-phosphaphenanthrene-10-oxide derivative(DiDOPO)	5	35	491	81	2.29	35.8	V-0	[31]
phenethyl-bridged 9,10-dihydro-9-oxa-10-phosphaphenanthrene-10-oxide derivative (DiDOPO)	10	38	508	83	2.35	38	V-0	[31]
	0	32	781	107	—	21.8	NR	[32]
phenethyl-bridged 9,10-dihydro-9-oxa-10-phosphaphenanthrene-10-oxide derivative (DiDOPO)	1	33	516	116	1.43	24.1	V-2	[32]
phenethyl-bridged 9,10-dihydro-9-oxa-10-phosphaphenanthrene-10-oxide derivative (DiDOPO)	5	35	491	81	2.29	35.7	V-0	[32]
phenethyl-bridged 9,10-dihydro-9-oxa-10-phosphaphenanthrene-10-oxide derivative (DiDOPO)	10	38	508	83	2.35	38	V-0	[32]
phenethyl-bridged 9,10-dihydro-9-oxa-10-phosphaphenanthrene-10-oxide derivative (DiDOPO)	15	41	436	72	3.41	33.6	V-0	[32]
phenethyl-bridged 9,10-dihydro-9-oxa-10-phosphaphenanthrene-10-oxide derivative (DiDOPO)	20	16	298	68	2.06	27.5	V-0	[32]
	0	19	1324.6	95.7	—	19.2	HB	[33]
pentaerythritol phosphate melamine salt (PPMS)	15	20	491.6	74	3.66	22.8	V-2	[33]
pentaerythritol phosphate melamine salt functionalized Expandable graphite (PPMS-EG)	15	16	414.3	66.7	3.86	25.8	V-1	[33]
	0	15	1334.6	100.1	—	19.3	HB	[34]
Pentaerythritol phosphate melamine salt-functionalized Multiwalled carbon nanotube (PPMS-MWCNT)	5	13	1013.4	93.7	1.21	21.5	HB	[34]
Pentaerythritol phosphate melamine salt-functionalized Multiwalled carbon nanotube (PPMS-MWCNT)	10	8	680.7	90.7	1.15	22.6	V-2	[34]
Pentaerythritol phosphate melamine salt-functionalized Multiwalled carbon nanotube (PPMS-MWCNT)	15	6	444.6	77.6	1.54	24.5	V-2	[34]
pentaerythritol phosphate melamine salt (PPMS)	15	11	489.5	85.2	2.34	22.8	V-2	[34]
	0	66	793.5	86.3	—	21	NR	[35]
diphenyl 1H-imidazol-1-ylphosphonate (DPIPP)	7.5	56	535.2	61.3	1.77	27.5	NR	[35]
diphenyl 1H-imidazol-1-ylphosphonate (DPIPP)	15	59	427.5	53.7	2.66	31.5	V-0	[35]
1-(diphenylphosphinyl)-1H-imidazole oxide (DPPIO)	7.5	62	583.1	60	1.83	33	NR	[35]
1-(diphenylphosphinyl)-1H-imidazole oxide (DPPIO)	15	63	432.9	48.4	3.11	38	V-0	[35]
	0	57	770.1	82.6	—	20.5	NR	[36]
imidazolium dibenzo[c,e[1,2]oxaphosphate (IDOP)	5	65	617.5	65.8	1.78	27	NR	[36]
imidazolium dibenzo[c,e [1,2]oxaphosphate (IDOP)	10	67	586.5	64.2	1.98	34.5	V-1	[36]
imidazolium dibenzo[c,e [1,2]oxaphosphate (IDOP)	15	68	485.6	51.2	3.05	37	V-0	[36]
	0	63	731.2	103.2	—	21.1	NR	[37]
polyphosphoric acid piperazine (PPAP)	5	38	511.9	92.5	0.96	30.8	V-0	[37]
diglycidyl ether of bisphenol A epoxy resin epoxy/hollow glass microspheres(foam)	0	17	444.92	138.2	—	21.5	NR	[38]
aluminum diisobutylphosphinate (AlPBu)	10	17	272.28	113.2	1.99	26.5	NR	[38]
aluminum diisobutylphosphinate (AlPBu)	12.5	17	264.98	110.8	2.09	27.8	V-1	[38]
Aluminum diisobutylphosphinate (AlPBu)	15	17	260.77	109.3	2.15	29	V-0	[38]
	0	53	1484	86.4	—	26	NR	[39]
6-morpholino-6Hdibenzo[c,e][1,2]oxaphosphinine 6-oxide (MPL-DOPO)	2.5	46	1296	74.3	1.15	29.5	V-1	[39]
6-morpholino-6Hdibenzo[c,e][1,2]oxaphosphinine 6-oxide (MPL-DOPO)	5	45	1145	67.1	1.41	30.5	V-0	[39]
6,6′-((methylenebis(4,1 phenylene))bis(azanediyl))bis(6Hdibenzo[c,e][1,2]oxaphosphinine 6-oxide) (DDM-DOPO)	2.5	51	1236	76.5	1.30	30	V-0	[39]
6,6′-((methylenebis(4,1 phenylene))bis(azanediyl))bis(6Hdibenzo[c,e][1,2]oxaphosphinine 6-oxide) (DDM-DOPO)	5	48	999	69.7	1.66	31.5	V-0	[39]
	0	71	654.3	100.3	—	25.7	NR	[40]
6-(((1H-tetrazol-5-yl)amino)(4hydroxyphenyl)methyl)dibenzo[c,e][1,2]oxaphosphinine 6-oxide (ATZ)	6	81	482.5	83.9	1.84	33.7	V-0	[40]
Waterborne EP resin	0	25	343.7	18.3	—	19.3	NR	[41]
phosphated K-carrageenan (P-KC)	30	14	313.7	19.3	0.58	20.8	NR	[41]
9,10-dihydro-9-oxa-10-phosphaphenanthrene-10-oxide (DOPO)	30	10	279.6	15.1	0.59	22.1	V-1	[41]
	0	39	1162	104	—	26.8	NR	[42]
Tris(Bis(4((Diphenoxyphosphoryl)Oxy)Phenyl)Methyl)Benzene-1,3,5-Tricarboxylate (DHPP-OH-BAC)	5	50	796	97	2.00	31.2	V-2	[42]
Tris(Bis(4((Diphenoxyphosphoryl)Oxy)Phenyl)Methyl)Benzene-1,3,5-Tricarboxylate (DHPP-OH-BAC)	10	58	643	91	3.07	32.4	V-1	[42]
Tris(Bis(4((Diphenoxyphosphoryl)Oxy)Phenyl)Methyl)Benzene-1,3,5-Tricarboxylate (DHPP-OH-BAC)	15	62	610	88	3.57	33.6	V-0	[42]
	0	40	1511.7	115.8	—	19	NR	[43]
poly(pentaerythritol phosphate phosphinic acyl piperazine) (PPAP)	5	38	838.1	75.4	2.63	26	NR	[43]
poly(pentaerythritol phosphate phosphinic acyl piperazine) (PPAP)	10	36	522	54.2	5.56	28	V-1	[43]
poly(pentaerythritol phosphate phosphinic acyl piperazine) (PPAP)	20	34	416	44.5	8.03	35	V-0	[43]
	0	61	1125.8	66.2	—	26.5	NR	[44]
1-methyl-3-((6-oxidodibenzo[c,e][1,2]oxaphosphinin 6-yl)methyl)-1H-imidazol-3-ium 4 methylbenzenesulfonate ([Dmim]Tos)	2.4	51	947.6	67.3	0.97	31.7	V-1	[44]
1-methyl-3-((6-oxidodibenzo[c,e][1,2]oxaphosphinin 6-yl)methyl)-1H-imidazol-3-ium 4 methylbenzenesulfonate ([Dmim]Tos)	4	57	705.4	57.6	1.71	32.5	V-0	[44]
1-methyl-3-((6-oxidodibenzo[c,e][1,2]oxaphosphinin 6-yl)methyl)-1H-imidazol-3-ium 4 methylbenzenesulfonate ([Dmim]Tos)	7.5	51	767	56.2	1.44	33.9	V-0	[44]
	0	32	1111	18.2	—	20.5	NR	[45]
melamine phenylphosphate (MPhP)	10	38	1008	12.4	1.92	23.5	NR	[45]
melamine phenylphosphate (MPhP)	15	40	846	12.2	2.44	24.5	V-1	[45]
melamine phenylphosphate (MPhP)	20	41	545	12	3.96	26.5	V-0	[45]
	0	74	1205.4	77.1	—	26.4	NR	[46]
melamine-organophosphinic acid salt (MDOP)	0.96	79	1426.4	75.4	0.92	31	V-1	[46]
melamine-organophosphinic acid salt (MDOP)	1.9	76	1209.5	74.2	1.06	32	V-1	[46]
melamine-organophosphinic acid salt (MDOP)	3.75	78	915.3	67.1	1.59	35.6	V-0	[46]
melamine-organophosphinic acid salt (MDOP)	7.24	67	660.7	60.2	2.11	38	V-0	[46]
	0	70	1491	81	—	19	NR	[47]
aluminum diethyl phosphinate (AlPi)	7	58	572	63	2.77	28.5	V-0	[47]
Melamine polyphosphate (MPP)	7	75	479	68	3.97	—	—	[47]
	0	70	1000.5	95.2	—	22.6	NR	[48]
bisphenol-A bridged penta(phenoxy)cyclotriphosphazene (A-BP)	9	62	783	55.9	1.92	33.9	V-0	[48]
	0	60	1285	83.5	—	25.5	NR	[19]
cage–ladder-structure, phosphorus-containing polyhedral oligomeric silsesquinoxane (CLEP–DOPO–POSS) via the hydrolytic condensation of 9,10-dihydro-9-oxa-10-phosphaphenanthrene-10-oxide (DOPO)–vinyl trimethoxysilane (VTMS)with 2-(3,4-epoxycyclohexyl) ethyl trimethoxysilane (CLEP–DOPO–POSS)	2.91	62	961	84.9	1.35	31.9	V-0	[19]
	0	95	939	98	—	23	NR	[49]
copper phenylphosphate nanoplate (CuPP)	1	103	511	93	2.09	32.4	NR	[49]
copper phenylphosphate nanoplate (CuPP)	2	80	466	83	2.00	35.5	V-1	[49]
copper phenylphosphate nanoplate (CuPP)	4	88	454	82	2.28	38.2	V-1	[49]
copper phenylphosphate nanoplate (CuPP)	6	88	448	72	2.64	37.8	V-1	[49]
copper phenylphosphate nanoplate (CuPP)	8	86	401	73	2.84	34.6	V-1	[49]
	0	69	1139.7	75.7	—	25.2	NR	[50]
reaction of 2-chloro-5,5-dimethyl-1,3,2-dioxaphosphinane-2-oxide & 2-aminobenzothiazole (DOP-ABZ)	15	66	327.2	63	4.00	26.8	V-1	[50]
reaction of 2-chloro-5,5-dimethyl-1,3,2-dioxaphosphinane-2-oxide & 2-aminobenzothiazole (DOP-ABZ)	17.5	65	308.9	40.6	6.48	27.5	V-0	[50]
reaction of 2-chloro-5,5-dimethyl-1,3,2-dioxaphosphinane-2-oxide & 2-aminobenzothiazole (DOP-ABZ)	20	52	238.9	28	9.72	28.3	V-0	[50]
	0	36	1558	93	—	24.2	NR	[51]
9,10-Dihydro-9-oxa-10-phosphaphenanthrene-10-oxide (DOPO)	7.11	33	1301	64.6	1.58	35.1	V-1	[51]
reaction between 1,4-Phthalaldehyde & 2-benzothiazolamine & 9,10-Dihydro-9-oxa-10-phosphaphenanthrene-10-oxide (BPD)	3.38	34	1313	78.9	1.32	32.8	V-1	[51]
reaction between 1,4-Phthalaldehyde & 2-benzothiazolamine & 9,10-Dihydro-9-oxa-10-phosphaphenanthrene-10-oxide (BPD)	6.71	32	1273	69.8	1.44	34.3	V-1	[51]
reaction between 1,4-Phthalaldehyde & 2-benzothiazolamine & 9,10-Dihydro-9-oxa-10-phosphaphenanthrene-10-oxide (BPD)	10.04	33	1220	63.8	1.70	36.9	V-0	[51]
reaction between 1,4-Phthalaldehyde & 2-benzothiazolamine & 9,10-Dihydro-9-oxa-10-phosphaphenanthrene-10-oxide (BPD)	13.41	31	1071	59.1	1.97	39.1	V-0	[51]
	0	61	1208	77.3	—	22.5	NR	[52]
9,10-Dihydro-9-oxa-10-phosphaphenanthrene-10-oxide (DOPO)	7.7	56	828	61.6	1.68	34.5	V-1	[52]
hexa-phenoxy-cyclotriphosphazene (HPCP)	8.2	52	510	63.1	2.47	32.5	V-1	[52]
		78	1934.2	103.3	—	23.5	NR	[53]
reaction between 4-(hydroxymethyl)-2,6,7-trioxa-1-phosphabicyclo[2.2.2]octane 1-oxide & 6-(2,5-dihydroxyphenyl)-6H-dibenzo[c,e][1,2]oxaphosphinine 6-oxide (DOPO-TPMP)	2.5	76	1683.9	91.1	1.26	28.2	V-1	[53]
reaction between 4-(hydroxymethyl)-2,6,7-trioxa-1-phosphabicyclo[2.2.2]octane 1-oxide & 6-(2,5-dihydroxyphenyl)-6H-dibenzo[c,e] [1,2]oxaphosphinine 6-oxide (DOPO-TPMP)	5	72	1544.8	82.9	1.44	34.8	V-1	[53]
reaction between 4-(hydroxymethyl)-2,6,7-trioxa-1-phosphabicyclo[2.2.2]octane 1-oxide & 6-(2,5-dihydroxyphenyl)-6H-dibenzo[c,e][1,2]oxaphosphinine 6-oxide (DOPO-TPMP)	7.5	72	1483.6	75.7	1.64	35.6	V-0	[53]
reaction between 4-(hydroxymethyl)-2,6,7-trioxa-1-phosphabicyclo[2.2.2]octane 1-oxide & 6-(2,5-dihydroxyphenyl)-6H-dibenzo[c,e][1,2]oxaphosphinine 6-oxide (DOPO-TPMP)	10	63	819.3	69.2	2.84	36.1	V-0	[53]
		54	880	187	—	24.1	NR	[54]
10-(hydroxy(4-hydroxyphenyl)methyl)-5,10-dihydrophenophosphazinine-10-oxide (HB-DPPA)	2	65	800	162	1.52	29.3	V-0	[54]
		53	1121	102	—	20	NR	[55]
ammonium polyphosphate (APP)	21	57	594	53	3.90	33	NR	[55]
ethanediamine-modified ammonium polyphosphate (EDA-APP)	21	61	398	54	6.12	33	V-0	[55]
		45	1091	83	—	22.8	NR	[56]
hexakis(4-boronic acid-phenoxy)-cyclophosphazene (CP-6B)	3	42	608	71	1.95	30.8	V-0	[56]
		57	1108	96.2	—	22	NR	[57]
N,N′-diamyl-p-phenylphosphonicdiamide (PM)	2	56	970	84.2	1.28	24.5	NR	[57]
N,N′-diamyl-p-phenylphosphonicdiamide (PM)	6	54	840	78.5	1.53	25.5	NR	[57]
IC: inclusion complex β-cyclodextrin & N,N′-diamyl-p-phenylphosphonicdiamide (PM-βCD)	2	55	905	73	1.55	26.5	NR	[57]
IC: inclusion complex β-cyclodextrin & N,N′-diamyl-p-phenylphosphonicdiamide (PM-βCD)	6	50	541	68.8	2.51	26.8	NR	[57]
		43	469	66.2	—	24.7	NR	[58]
poly(4,40-diamino diphenyl sulfone 2,6,7-trioxa-1-phosphabicyclo[2.2.2]octane-4-methanol-substituted phosphoramide) (PSA)	10	28	149	33.2	4.08	28	V-1	[58]
poly(4,40-diamino diphenyl sulfone 2,6,7-trioxa-1-phosphabicyclo[2.2.2]octane-4-methanol-substituted phosphoramide) (PSA)	20	26	118	21.7	7.33	31	V-0	[58]
		82	1148	88.4	—	21	NR	[59]
bisphenol A bridged penta(anilino) cyclotriphosphazene (BPA-BPP)	9	72	457	78.4	2.48	28.7	V-1	[59]
		46	1291	87.2	—	23	NR	[60]
9,10-dihydro-9-oxa-10-phosphaphenanthrene-10-oxide (DOPO)	9.1	26	893	59.6	1.19	29	NR	[60]
1-oxo-4-hydroxymethyl-2,6,7-trioxa-l phosphabicyclo[2.2.2] octane (PEPA)	9.1	40	847	59.5	1.94	28	NR	[60]
reaction between 9,10-dihydro-9-oxa-10-phosphaphenanthrene-10-oxide-1-oxo-4-hydroxymethyl-2,6,7-trioxa-l phosphabicyclo[2.2.2] octane (DOPO-PEPA)	5.7	44	873	60.9	2.02	30	V-0	[60]
reaction between 9,10-dihydro-9-oxa-10-phosphaphenanthrene-10-oxide-1-oxo-4-hydroxymethyl-2,6,7-trioxa-l phosphabicyclo[2.2.2] octane (DOPO-PEPA)	7.4	48	683	46.3	3.71	35	V-0	[60]
reaction between 9,10-dihydro-9-oxa-10-phosphaphenanthrene-10-oxide-1-oxo-4-hydroxymethyl-2,6,7-trioxa-l phosphabicyclo[2.2.2] octane (DOPO-PEPA)	9.1	42	595	45.9	3.76	35	V-0	[60]
		58	839	129	—	—	NR	[61]
polyhedral oligomeric silsesquioxane containing 9,10-dihydro-9-oxa-10 phosphaphenanthrene-10-oxide (DOPO-POSS)	2.5	58	631	104	1.64	27.1	V-1	[61]
polyhedral oligomeric silsesquioxane containing 9,10-dihydro-9-oxa-10 phosphaphenanthrene-10-oxide (DOPO-POSS)	5	62	404	87	3.29	—	NR	[61]
polyhedral oligomeric silsesquioxane containing 9,10-dihydro-9-oxa-10 phosphaphenanthrene-10-oxide (DOPO-POSS)	10	61	346	79	4.16	—	NR	[61]
		53	1034	114	—	24.2	NR	[62]
Hexaphenoxycyclotriphosphazene (HPCTP)	7.46	56	918	94	1.44	26.2	V-1	[62]
Hexaphenoxycyclotriphosphazene (HPCTP)	11.19	53	796	83	1.78	28	V-0	[62]
Hexaphenoxycyclotriphosphazene (HPCTP)	14.92	54	840	78	1.83	28.6	V-0	[62]
9,10-dihydro-9-oxa-10-phosphaphenanthrene-10-oxide (DOPO)	6.97	51	947	92	1.30	25.9	NR	[62]
9,10-dihydro-9-oxa-10-phosphaphenanthrene-10-oxide (DOPO)	10.46	50	850	88	1.48	27.4	NR	[62]
9,10-dihydro-9-oxa-10-phosphaphenanthrene-10-oxide (DOPO)	13.94	46	785	81	1.60	27.8	V-1	[62]
		60	872.8	88.5	—	22.5	NR	[63]
2-(hydroxy(phenyl)methyl)-5,5-dimethyl-1,3,2-dioxaphosphinane 2-oxide (TP)	12.42	23	312.6	59	1.60	31.8	V-1	[63]
[4-(2,4,6-Tris[24] dioxaphosphinan-2-yl) hydroxymety] phenoxy]-(1,3,5)-triazine (TNTP)	14.36	34	253	65.8	2.62	32.4	V-0	[63]
		47	1208	81	—	22.5	NR	[64]
9,10-dihydro-9-oxa-10 phosphaphenanthrene-10-oxide (DOPO)	7	32	853	64	1.22	34	V-1	[64]
reaction between triglycidyl isocyanurate, 9,10-dihydro-9-oxa-10-phosphaphenanthrene-10-oxide & phenylboronic acid (BNP)	7	38	505	60	2.61	29.5	NR	[64]
reaction between triglycidyl isocyanurate, 9,10-dihydro-9-oxa-10-phosphaphenanthrene-10-oxide & phenylboronic acid (BNP)	11	35	425	52	3.29	32	V-1	[64]
reaction between triglycidyl isocyanurate, 9,10-dihydro-9-oxa-10-phosphaphenanthrene-10-oxide & phenylboronic acid (BNP)	14.7	34	410	50	3.45	32.5	V-0	[64]
reaction between triglycidyl isocyanurate, 9,10-dihydro-9-oxa-10-phosphaphenanthrene-10-oxide & phenylboronic acid (BNP)	18.4	33	400	47	3.65	33.3	V-0	[64]
		47	1208	81	—	22.5	NR	[65]
9,10-dihydro-9-oxa-10 phosphaphenanthrene-10-oxide (DOPO)	7	32	853	64	1.22	34	V-1	[65]
reaction between triglycidyl isocyanurate & 9,10-dihydro-9-oxa-10 phosphaphenanthrene-10-oxide & boric acid (DTB)	7	32	556	61	1.96	31.5	NR	[65]
reaction between triglycidyl isocyanurate & 9,10-dihydro-9-oxa-10 phosphaphenanthrene-10-oxide & boric acid (DTB)	10	33	453	55	2.75	33.2	V-1	[65]
reaction between triglycidyl isocyanurate & 9,10-dihydro-9-oxa-10 phosphaphenanthrene-10-oxide & boric acid (DTB)	15	34	425	54	3.08	35.6	V-0	[65]
reaction between triglycidyl isocyanurate & 9,10-dihydro-9-oxa-10 phosphaphenanthrene-10-oxide & boric acid (DTB)	20	31	461	57	2.45	35.2	V-0	[65]
		58	1208	80.6	—	22.5	NR	[66]
9,10-dihydro-9-oxa-10-phosphaphenanthrene-10-oxide (DOPO)	7.7	58	828	63.7	1.84	34.5	V-1	[66]
hexa-phenoxy-cyclotriphosphazene (HPCP)	8.2	49	510	64	2.52	32.5	V-1	[66]
		57	1557	94.5	—	24.5	NR	[67]
9,10-dihydro-9-oxa-10-phosphaphenanthrene-10-oxide (DOPO)	7.1	52	1301	65	1.58	35.2	V-1	[67]
		61	1208	80.6	—	22.5	NR	[68]
9,10-dihydro-9-oxa-10-phosphaphenanthrene-10-oxide (DOPO)	7	58	833	66.3	1.67	34	V-1	[68]
tri(phosphaphenanthrene-maleimide-phenoxyl)-triazine (DOPO-TMT)	7	56	919	71.2	1.36	29.5	NR	[68]
tri(phosphaphenanthrene-maleimide-phenoxyl)-triazine (DOPO-TMT)	10.4	56	694	63.7	2.02	33	V-1	[68]
tri(phosphaphenanthrene-maleimide-phenoxyl)-triazine (DOPO-TMT)	13.9	53	776	60.6	1.79	36.2	V-0	[68]
tri(phosphaphenanthrene-maleimide-phenoxyl)-triazine (DOPO-TMT)	17.3	48	556	56.5	2.43	37.5	V-0	[68]
tri(phosphaphenanthrene-maleimide-phenoxyl)-triazine (DOPO-TMT)	20.8	50	674	59.6	1.98	38.4	V-0	[68]
		47	1208	80.6	—	22.5	NR	[69]
hexa(4-maleimido-phenoxyl) cyclotriphosphazene (HMCP)	3.4	39	751	77	1.39	27	NR	[69]
hexa(4-maleimido-phenoxyl) cyclotriphosphazene (HMCP)	6.8	38	469	66.5	2.52	29	V-1	[69]
hexa(4-maleimido-phenoxyl) cyclotriphosphazene (HMCP)	10.2	36	506	63	2.33	33.4	V-0	[69]
hexa(4-maleimido-phenoxyl) cyclotriphosphazene (HMCP)	13.6	36	467	58	2.75	35	V-0	[69]
hexa(4-maleimido-phenoxyl) cyclotriphosphazene (HMCP)	17	39	351	50	4.60	36.5	V-0	[69]
		53	939.2	227.4	—	24.2	NR	[70]
addition reaction between DOPO and Schiff-base obtained in advance by the condensation of 4,4′-diaminodiphenyl methane & 4-hydroxybenzaldehyde (DOPO-bp)	3.4	48	757.1	154.1	1.65	30.5	V-1	[70]
addition reaction between DOPO and Schiff-base obtained in advance by the condensation of 4,4′-diaminodiphenyl methane & 4-hydroxybenzaldehyde (DOPO-bp)	6.7	47	633.9	145.2	2.05	39.7	V-0	[70]
addition reaction between DOPO and Schiff-base obtained in advance by the condensation of 4,4′-diaminodiphenyl methane & 4-hydroxybenzaldehyde (DOPO-bp)	13.5	39	535.1	121.9	2.40	41.6	V-0	[70]
		63	619.9	77.6	—	21.7	NR	[71]
hexa-[4-(phydroxyanilino- phosphaphenanthrene methyl)-phenoxyl]-cyclotriphosphazene (CTP-DOPO)	10.6	52	349.9	51.7	2.19	36.6	V-0	[71]
		63	731.2	103.2	—	20.3	NR	[72]
polymelamine tetramethylene phosphonium sulfate (PMTMPS)	11	59	489.9	80.9	1.78	32.5	V-0	[72]
		63	731.4	103.2	—	20.3	NR	[73]
poly(urea tetramethylene phosphonium sulfate) (PUTMPS)	12	57	525.8	79.2	1.63	31.3	V-0	[73]
		56	1420	144	—	26.2	NR	[74]
aluminum poly-hexamethylenephosphinate (APHP)	2	54	742	98	2.71	29.3	NR	[74]
aluminum poly-hexamethylenephosphinate (APHP)	4	58	540	95	4.12	32.7	V-1	[74]
aluminum poly-hexamethylenephosphinate (APHP)	6	55	603	93	3.58	33.1	NR	[74]
		56	1420	116	—	26.2	NR	[75]
aluminum poly-hexamethylenephosphinate (APHP)	6	55	603	69	3.88	33.1	NR	[75]
9,10-dihydro-9-oxa-10-phosphaphenanthrene 10-oxide (DOPO)	6	44	725	70	2.55	38.5	V-1	[75]
		101	685	106	—	19	NR	[76]
α,ω-dicarboxyl aromatic polyphosphonate (HP-1001-COOH)	10	72	454	84	1.35	26.6	NR	[76]
α,ω-dicarboxyl aromatic polyphosphonate (HP-1001-COOH)	20	68	393	79	1.57	30.9	NR	[76]
α,ω-dicarboxyl aromatic polyphosphonate (HP-1001-COOH)	30	66	324	75	1.95	32.4	V-0	[76]
α,ω-dicarboxyl aromatic polyphosphonate (HP-1001-COOH)	40	68	351	74	1.88	30.3	V-0	[76]
α,ω-dicarboxyl aromatic polyphosphonate (HP-1001-COOH)	50	76	351	85	1.83	27	V-1	[76]
		56	1420	140	—	26	NR	[77]
reaction between triallyl isocyanurate & 9,10-dihydro-9-oxa-10-phosphaphenanthrene-10-oxide (TAD)	4	46	1106	82	1.80	33.6	V-1	[77]
		69	966	93.9	—	22.5	NR	[78]
9,10-dihydro-9-oxa-10-phosphaphenanthrene-10-oxide (DOPO)	10	50	463	64.8	2.19	30.6	V-1	[78]
reaction between triallyl isocyanurate & 9,10-dihydro-9-oxa-10-phosphaphenanthrene-10-oxide (TAD)	6	51	691	60.8	1.59	32.4	NR	[78]
reaction between triallyl isocyanurate & 9,10-dihydro-9-oxa-10-phosphaphenanthrene-10-oxide (TAD)	8	56	590	53.7	2.32	32.6	V-1	[78]
reaction between triallyl isocyanurate & 9,10-dihydro-9-oxa-10-phosphaphenanthrene-10-oxide (TAD)	10	54	452	57.7	2.72	34.2	V-1	[78]
reaction between triallyl isocyanurate & 9,10-dihydro-9-oxa-10-phosphaphenanthrene-10-oxide (TAD)	12	55	641	55.7	2.02	33.5	V-0	[78]
		52	1334.3	58.8	—	22.2	NR	[79]
piperazine-modified ammonium polyphosphate (PAz-APP)	10	33	261.5	15.6	12.20	29	V-0	[79]
piperazine-modified ammonium polyphosphate (PAz-APP)	15	33	246.1	11.3	17.90	31.5	V-0	[79]
		40	980.4	55.2	—	21.5	NR	[80]
diethylenetriamine-modified ammonium polyphosphate (DETA-APP)	10	35	388	12.7	9.60	28.5	V-0	[80]
diethylenetriamine-modified ammonium polyphosphate (DETA-APP)	15	32	310.5	11.4	12.23	30.5	V-0	[80]
		52	995	93.3	—	22.5	NR	[81]
9,10-dihydro-9-oxa-10-phosphaphenanthrene-10-oxide (DOPO)	8.3	57	437.2	60.6	3.84	31.7	V-1	[81]
tri-(phosphaphenanthrene-(hydroxyl-methylene)-phenoxyl)-1, 3, 5-triazine (Trif-DOPO)	11.7	48	390.8	70.4	3.11	33.9	NR	[81]
tri-(phosphaphenanthrene-(hydroxyl-methylene)-phenoxyl)-1, 3, 5-triazine (Trif-DOPO)	14	44	420.7	67.9	2.74	36	V-0	[81]
		61	1420	144	—	26.4	NR	[82]
addition reaction of 1,3,5-triglycidyl isocyanurate & 9,10-dihydro-9-oxa-10-phosphaphenanthrene-10-oxide & 10-(2,5-dihydroxyphenyl)-10-H-9-oxa-10-phosphaphenanthrene-10-oxide (TOD)	2	61	852	89	2.69	32.8	V-1	[82]
addition reaction of 1,3,5-triglycidyl isocyanurate & 9,10-dihydro-9-oxa-10-phosphaphenanthrene-10-oxide & 10-(2,5-dihydroxyphenyl)-10-H-9-oxa-10-phosphaphenanthrene-10-oxide (TOD)	4	61	830	77	3.19	35.9	V-0	[82]
addition reaction of 1,3,5-triglycidyl isocyanurate & 9,10-dihydro-9-oxa-10-phosphaphenanthrene-10-oxide & 10-(2,5-dihydroxyphenyl)-10-H-9-oxa-10-phosphaphenanthrene-10-oxide (TOD)	6	61	720	69	4.11	38	V-0	[82]
		68	1730	110	—	23	NR	[83]
9,10-dihydro-9-oxa-10-phosphaphenanthrene-10-oxide-4,4-diaminodiphenyl methane (DOPO-DDM)	10	76	1480	49	2.93	29.5	V-1	[83]
9,10-dihydro-9-oxa-10-phosphaphenanthrene-10-oxide-4,4-diaminodiphenyl sulfone (DOPO-DDE)	10	78	1370	56	2.84	31.5	V-0	[83]
9,10-dihydro-9-oxa-10-phosphaphenanthrene-10-oxide-4,40-diaminodiphenyl ether (DOPO-DDS)	10	74	1190	60	2.90	31	V-0	[83]
		61	893	112	—	23	NR	[84]
diphenylphosphine containing polyhedral oligomeric silsesquioxanes (DPP-POSS)	5	65	489	94.1	2.31	33.2	V-0	[84]
diphenylphosphine oxide containing polyhedral oligomeric silsesquioxanes (DPOP-POSS)	5	62	419	87.8	2.76	29.3	V-1	[84]
9,10-dihydro-9-oxa-10-phosphaphenanthrene-10-oxide containing polyhedral oligomeric silsesquioxanes (DOPO-POSS)	5	64	433	91.1	2.66	30	V-1	[84]
		69	961	96	—	20	NR	[85]
9,10-dihydro-9-oxa-10-phosphaphenanthrene-10-oxide modified Aluminum hydroxide (ATH-DOPO)	10	75	586	64	2.67	25.6	NR	[85]
9,10-dihydro-9-oxa-10-phosphaphenanthrene-10-oxide modified Aluminum hydroxide (ATH-DOPO)	20	87	341	57	5.98	27.7	V-0	[85]
9,10-dihydro-9-oxa-10-phosphaphenanthrene-10-oxide modified honeycomb-like mesoporous aluminum hydroxide (pATH-DOPO)	10	75	391	52	4.93	27.1	V-0	[85]
		70	1000	89	—	21.5	NR	[86]
bisphenol-S bridged penta(anilino)cyclotriphosphazene (BPS-BPP)	9	62	537	76	1.93	29.7	V-1	[86]
		62	688	106	—	21	NR	[87]
1,3,5-tris(3-(diphenylphosphoryl)propyl)-1,3,5-triazinane-2,4,6-trione (PN)	15	55	567	82	1.39	33.5	V-0	[87]
[(1,1,3,3-tetramethyl-1,3-disiloxanediyl)-di-2,1-ethanediyl]-bis(diphenylphosphine oxide) (PSi)	25	49	309	74	2.52	34	V-0	[87]
		75	685	95	—	20.3	NR	[88]
bis(2,6-dimethyphenyl) phenylphosphonate (BDMPP)	14	65	528	68	1.57	33.8	V-0	[88]
		62	840	84	—	23	V-1	[89]
amine-terminated cyclophosphazene (ATCP)	15	66	658	62	1.84	35	V-0	[89]
		57	713	64	—	28	V-1	[90]
amine-terminated cyclophosphazene (ATCP)	15	52	610	58	1.17	34	V-0	[90]
		63	1068	76	—	26	NR	[91]
9, 10-Dihydro-9-oxa-10-phosphaphenanthrene-10-oxide (DOPO)	4.5	83	724	73	2.02	31.5	V-1	[91]
reaction between 9, 10-Dihydro-9-oxa-10-phosphaphenanthrene-10-oxide & 2-aminobenzothiazole (DOPO-ABZ)	7.5	71	652	72	1.94	33.5	V-0	[91]
reaction between 9, 10-Dihydro-9-oxa-10-phosphaphenanthrene-10-oxide & 2-aminobenzothiazole (DOPO-ABZ)	10	66	609	67	2.08	33.5	V-0	[91]
		47	1208	81	—	22.5	NR	[92]
reaction between maleimide & phosphaphenanthrene & triazine-trione (DMT)	3.3	39	837	67	1.44	31.2	NR	[92]
reaction between maleimide & phosphaphenanthrene & triazine-trione (DMT)	6.6	35	685	63	1.68	32.8	NR	[92]
reaction between maleimide & phosphaphenanthrene & triazine-trione (DMT)	10	37	544	62	2.28	34.4	V-1	[92]
reaction between maleimide & phosphaphenanthrene & triazine-trione (DMT)	13.5	36	506	60	2.46	35.8	V-0	[92]
reaction between maleimide & phosphaphenanthrene & triazine-trione (DMT)	17	34	491	58	2.48	33	V-0	[92]
		50	860	112	—	23	NR	[93]
Ammonium polyphosphate (APP)	10	59	458	62	4.00	25	NR	[93]
Ammonium polyphosphate–montmorillonite (APP-MMT)	10	60	393	34	8.65	30	V-0	[93]
		50	860	133	—	23	NR	[94]
9,10-dihydro-9-oxa-10-phosphaphenanthrene-10-oxide (DOPO)	6	64	502	79	3.69	31.2	V-1	[94]
9,10-dihydro-9-oxa-10-phosphaphenanthrene-10-oxide-Montmorillonite (DOPO-MMT)	6	59	398	73	4.64	33.4	V-0	[94]
		65	966	96	—	22.5	NR	[95]
aluminum poly-hexamethylenephosphinate (APHP)	10	56	855	90	1.03	31.5	NR	[95]
bisphenol-A bis(diphenyl phosphate) (BDP)	10	50	746	86	1.11	33.4	NR	[95]
		56	722.7	86.7	—	20.5	NR	[96]
isopropylphenyl phosphate (FIPF)	20	47	363.1	61	2.37	33	V-0	[96]
tertbutylphenyl phosphate (FTBF)	20	50	361.8	61.4	2.51	30.3	V-0	[96]
		47	955	59.7	—	22.5	NR	[97]
phenylphosphonic di-benzothiazolyl amide (PPDAB)	10	65	611	46.4	2.78	31	V-0	[97]
		48	1227	111	—	26.8	NR	[98]
boron phosphate (BP)	5	46	892	91	1.60	28.3	V-1	[98]
boron phosphate (BP)	9	47	805	89	1.86	29.2	V-1	[98]
boron phosphate (BP)	15	46	602	84	2.58	31.5	V-1	[98]
		40	1163.1	90.3	—	22	NR	[99]
polystyrene encapsulating ammonium polyphosphate (PS-APP)	2	21	1092.2	86.4	0.58	23.2	NR	[99]
polystyrene encapsulating ammonium polyphosphate (PS-APP)	5	20	959.5	92.6	0.59	25.7	V-1	[99]
polystyrene encapsulating ammonium polyphosphate (PS-APP)	10	10	614.2	85.8	0.49	26.8	V-1	[99]
polystyrene encapsulating ammonium polyphosphate (PS-APP)	15	8	375.4	65.7	0.85	28.5	V-1	[99]
polystyrene encapsulating ammonium polyphosphate (PS-APP)	20	25	733.7	81.7	1.09	28.7	V-1	[99]
		46	892	137	—	20	NR	[100]
polyhedral oligomeric silsesquioxane containing 9,10-dihydro-9-oxa-10-phosphaphenanthrene-10-oxide (DOPO-POSS)	2.5	46	963	129	0.98	21.5	NR	[100]
polyhedral oligomeric silsesquioxane containing 9,10-dihydro-9-oxa-10-phosphaphenanthrene-10-oxide (DOPO-POSS)	5	47	937	128	1.04	23.5	NR	[100]
polyhedral oligomeric silsesquioxane containing 9,10-dihydro-9-oxa-10-phosphaphenanthrene-10-oxide (DOPO-POSS)	10	46	690	113	1.56	25.9	V-1	[100]
		58	839	129	—	22	NR	[100]
polyhedral oligomeric silsesquioxane containing 9,10-dihydro-9-oxa-10-phosphaphenanthrene-10-oxide (DOPO-POSS)	2.5	58	631	104	1.64	27.1	V-1	[100]
polyhedral oligomeric silsesquioxane containing 9,10-dihydro-9-oxa-10-phosphaphenanthrene-10-oxide (DOPO-POSS)	5	62	404	87	3.29	26.2	NR	[100]
polyhedral oligomeric silsesquioxane containing 9,10-dihydro-9-oxa-10-phosphaphenanthrene-10-oxide (DOPO-POSS)	10	61	346	79	4.16	24.8	NR	[100]
		45	855	112	—	25	NR	[101]
polyhedral oligomeric silsesquioxane containing 9,10-dihydro-9-oxa-10-phosphaphenanthrene-10-oxide (DOPO-POSS)	2.5	48	969	103	1.02	30.2	V-1	[101]
polyhedral oligomeric silsesquioxane containing 9,10-dihydro-9-oxa-10-phosphaphenanthrene-10-oxide (DOPO-POSS)	5	58	588	92	2.28	28.5	NR	[101]
polyhedral oligomeric silsesquioxane containing 9,10-dihydro-9-oxa-10-phosphaphenanthrene-10-oxide (DOPO-POSS)	10	61	483	85	3.16	23	NR	[101]
		45	855	112	—	25	NR	[102]
9,10-dihydro-9-oxa-10-phosphaphenanthrene-10-oxide (DOPO)	5	54	731	93	1.69	27.6	NR	[102]
		45	855	112	—	25	NR	[103]
9,10-dihydro-9-oxa-10-phosphaphenanthrene-10-oxide (DOPO)	6.3	54	686	96	1.74	30.5	NR	[103]
		45	855	112	—	25	NR	[104]
9,10-dihydro-9-oxa-10-phosphaphenanthrene-10-oxide (DOPO)	6.3	54	686	96	1.74	30.5	NR	[104]
		50	860	112	—	23	NR	[105]
ammonium polyphosphate montmorillonite nanocomposite (APP-MMT)	10	60	393	33	8.91	30	V-0	[105]
		50	860	112	—	23	NR	[106]
1-oxo-4-hydroxymethyl-2,6,7-trioxa-l-phosphabicyclo[2.2.2] octane (PEPA)	5.2	53	538	78	2.43	27	NR	[106]
Ammonium polyphosphate (APP)	2.9	61	1087	96	1.12	23.5	NR	[106]
9,10-dihydro-9-oxa-10-phosphaphenanthrene-10-oxide (DOPO)	6.3	55	684	76	2.03	32	NR	[106]
		76	1160.9	135	—	22.5	NR	[107]
poly(4,4-dihydroxy-1-methyl-ethyl diphenol-o-bicyclic pentaerythritol phosphatephosphate) (PCPBO)	5	65	882.8	132.1	1.14	27.3	NR	[107]
poly(4,4-dihydroxy-1-methyl-ethyl diphenol-o-bicyclic pentaerythritol phosphatephosphate) (PCPBO)	10	61	460.5	122.3	2.23	28.8	NR	[107]
poly(4,4-dihydroxy-1-methyl-ethyl diphenol-o-bicyclic pentaerythritol phosphatephosphate) (PCPBO)	15	44	375.4	119.8	2.017	30.3	V-1	[107]
poly(4,4-dihydroxy-1-methyl-ethyl diphenol-o-bicyclic pentaerythritol phosphatephosphate) (PCPBO)	20	31	337.1	117.3	1.616	31.2	V-0	[107]
		57	1730.27	114.16	—	21.5	NR	[108]
ammonium polyphosphate (APP)	15	63	397.89	35.49	15.46	36	V-0	[108]
glycidyl methacrylate microencapsulated ammonium polyphosphate (GMA-APP)	15	68	283.09	44	18.91	38.5	V-0	[108]
		62	1192	184	—	20.9	NR	[109]
ammonium polyphosphate(APP)	12	41	200	104	6.97	31	V-0	[109]
modified ammonium polyphosphate(MAPP)	12	47	184	98	9.22	32.5	V-0	[109]
		62	1192	184	—	20.9	NR	[110]
ammonium polyphosphate(APP)	12	41	200	104	6.97	31.9	V-0	[110]
		66	893	68	—	22.5	NR	[111]
hexa-(phosphaphenanthrene -hydroxyl-methyl-phenoxyl)-cyclotriphosphazene(HAP-DOPO)	9.3	51	383	53	2.31	31	V-0	[111]
hexa-(phosphaphenanthrene -hydroxyl-methyl-phenoxyl)-cyclotriphosphazene(HAP-DOPO)	15.47	43	303	41	3.18	30.8	V-0	[111]
		65	966	102	—	22.5	NR	[112]
ring-opening addition reaction between 1,3,5-triglycidyl isocyanurate & 9,10-dihydro-9-oxa-10-phosphaphenanthrene-10-oxide (TGIC-DOPO)	6.1	54	800	75	1.36	33.3	NR	[112]
ring-opening addition reaction between 1,3,5-triglycidyl isocyanurate & 9,10-dihydro-9-oxa-10-phosphaphenanthrene-10-oxide (TGIC-DOPO)	8.1	54	680	76	1.58	34.3	V-1	[112]
ring-opening addition reaction between 1,3,5-triglycidyl isocyanurate & 9,10-dihydro-9-oxa-10-phosphaphenanthrene-10-oxide (TGIC-DOPO)	10.2	50	520	71	2.05	35.2	V-1	[112]
ring-opening addition reaction between 1,3,5-triglycidyl isocyanurate & 9,10-dihydro-9-oxa-10-phosphaphenanthrene-10-oxide (TGIC-DOPO)	12.2	48	481	61	2.47	33.3	V-0	[112]
		35	1719	74.2	—	25	HB	[113]
9,10-dihydro-9-oxy-10-phosphaphenanthrene-10-oxide units linked to the star-shaped aliphatic ground body tetra-[(acryloyloxy)ethyl] pentarythrit (DOPP)	19.6	40	1191	44.8	2.73	37.9	V-1	[113]
9,10-dihydro-9-oxy-10-phosphaphenanthrene-10-oxide units linked to the star-shaped aliphatic ground body heterocyclic tris-[(acryloyloxy)ethyl] isocyanurate (DOPI)	23.1	36	869	41.5	3.63	34.2	V-0	[113]
		49	781	76	—	20.5	NR	[114]
poly(melamine-ethoxyphosphinyl-diisocyanate) (PMPC)	10	59	390	33	5.55	26	NR	[114]
poly(melamine-ethoxyphosphinyl-diisocyanate) (PMPC)	15	64	292	30	8.85	27.5	V-1	[114]
poly(melamine-ethoxyphosphinyl-diisocyanate) (PMPC)	20	59	235	27	11.26	28	V-0	[114]
		64	821	94	—	23.2	NR	[115]
9, 10-Dihydro-9-oxa-10-phosphaphenanthrene-10-oxide (DOPO)	5	54	461	70	2.01	33.7	V-1	[115]
		60	920	90.5	—	22.7	NR	[116]
((1,1,3,3-tetramethyldisiloxane-1,3-diyl)bis(propane-3,1-diyl))bis(2-methoxy-4,1-phenylene)bis(phenylphosphonochloridate) modified Magnesium-Aluminum layered double hydroxide (SIEPDP-Mg-Al LDH)	4	55	658	86.9	1.33	25.3	V-1	[116]
		64	939	179	—	19.6	NR	[117]
ammonium polyphosphate (APP)	5	61	283	111	5.09	27.1	V-0	[117]
		53	1262	84.7	—	25	NR	[118]
cardanol derived benzoxazine monomer (CBz)	10	49	1119	80.5	1.09	31	V-1	[118]
cardanol derived benzoxazine monomer (CBz)	15	50	920	79.4	1.38	32	V-0	[118]
cardanol derived benzoxazine monomer (CBz)	20	50	962	77.2	1.35	33	V-0	[118]
		59	1063	76.1	—	25.8	NR	[119]
poly (piperazine phosphaphenanthrene) (DOPMPA)	10	68	393	56.3	4.21	29	NR	[119]
poly (piperazine phosphaphenanthrene) (DOPMPA)	13	67	285	27.4	11.76	34	V-0	[119]
		27	673.7	56	—	22.3	NR	[9]
reaction of spirocyclic pentaerythritol bisphosphorate disphosphoryl chloride & 2,4-dihydroxybenzophenone (MFR)	10	26	402.3	53.3	1.69	29.6	V-1	[9]
reaction of spirocyclic pentaerythritol bisphosphorate disphosphoryl chloride & 2,4-dihydroxybenzophenone (MFR)	15	17	479.7	47.8	1.03	30.8	V-0	[9]
reaction of spirocyclic pentaerythritol bisphosphorate disphosphoryl chloride & 2,4-dihydroxybenzophenone (MFR)	20	22	241.6	42.3	3.00	32.2	V-0	[9]
		58	1369	135.6	—	23.5	NR	[17]
9,10-dihydro-9-oxa-10-phosphaphenanthrene-10-oxide-covalent organic frameworksnanosheets(reaction between melamine & o-phthalaldehyde) (DOPO-COFs)	0.4	70.2	1295	133.4	1.30	23.5	NR	[17]
9,10-dihydro-9-oxa-10-phosphaphenanthrene-10-oxide-covalent organic frameworksnanosheets(reaction between melamine & o-phthalaldehyde) (DOPO-COFs)	0.8	64	1086	125.3	1.50	24	NR	[17]
9,10-dihydro-9-oxa-10-phosphaphenanthrene-10-oxide-covalent organic frameworksnanosheets(reaction between melamine & o-phthalaldehyde) (DOPO-COFs)	1.6	58.6	1227	131.5	1.16	24.5	NR	[17]
9,10-dihydro-9-oxa-10-phosphaphenanthrene-10-oxide-covalent organic frameworksnanosheets(reaction between melamine & o-phthalaldehyde) (DOPO-COFs)	3.2	60.7	1117	110.5	1.57	25	NR	[17]
COFs: covalent organic frameworksnanosheets(reaction between melamine & o-phthalaldehyde) (COFs)	3.2	55	1295	140.4	0.96	24	NR	[17]
		21	1910	84.4	—	22.1	NR	[120]
melamine coated ammonium polyphosphate (Mel-APP)	20	22	312.6	30.8	17.54	32.6	V-0	[120]
	0	51	1914	81.9	—	22	NR	[121]
phosphorus and nitrogen-containing flame retardant (FR)	1	43	1631	69.6	1.16	22.5	NR	[121]
	0	50	1712	83.7	—	—	NR	[122]
poly(4,4′-diamino diphenyl sulfone phenyl phosphonamide) (ArPN_2_)	15	29	847	61.5	1.59	—	V-0	[122]
poly(bisphenol sulfone phenyl phosphonate) (ArPO_2_)	15	32	608	42.7	3.53	—	V-1	[122]
poly(4,4^′^-dia-minodiphenyl sulfone phenyl dichlorophosphate) (ArOPN_2_)	15.6	30	546	59.4	2.65	—	NR	[122]
poly(bisphenol sulfone phenoxy phosphate) (ArOPO_2_)	15.6	30	726	55.3	2.14	—	NR	[122]
	0	75	977	100	—	—	NR	[123]
ionic liquid-based metal–organic hybrid = Phosphomolybdic acid hydrate:PMA & 1-ethyl 3-(diethoxyphosphoryl)-propylimidazolium bromide:IL (PMAIL)	6	85	674.4	99	1.65	—	V-0	[123]
epoxy novolac resin	0	51	682	110	—	—	NR	[124]
oligo[DOPAc-2-tris(acryloyloxy)ethyl isocyanurate] (oDOPI)	13.81	52	426	86	2.08	—	V-0	[124]
Phosphazene (PZ)	10.8	50	466	80	1.97	—	V-0	[124]
melamine polyphosphate(MPP)	15	45	370	86	2.08	—	V-1	[124]
	0	50	985.7	91	—	—	NR	[125]
aluminum hypophosphite (AHP)	5	48	970.2	89	0.99	—	V-1	[125]
		23	1910	61	—	—	NR	[126]
Melamine coated ammonium polyphosphate (Mel-APP)	29.7	24	281	23	18.81	—	V-0	[126]
		54	1068	75.8	—	—	HB	[127]
Melamine poly(aluminum phosphate) (MPAlP)	20	40	540	60	1.85	—	HB	[127]
melamine poly(zinc phosphate) (MPZnP)	20	43	312	60	3.44	—	HB	[127]
melamine poly(magnesium phosphate) (MPMgP)	20	44	298	57.3	3.86	—	V-1	[127]
melamine polyphosphate (MPP)	20	38	244	26.6	8.77	—	V-0	[127]
diethyl aluminum phosphinate (AlPi-Et)	20	41	492	55.8	2.23	—	V-0	[127]
6H-dibenz[c,e][1,2] oxaphosphorin-6-propanoic acid, butyl ester, 6-oxide (DOPAc-Bu)	20	44	624	50.2	2.10	—	HB	[127]
		53	1084	115	—	—	NR	[128]
hexaphenoxycyclotriphosphazene (HPCTP)	5	58	807	96	1.76	—	V-0	[128]
hexaphenoxycyclotriphosphazene (HPCTP)	10	60	566	93	2.68	—	V-0	[128]
hexaphenoxycyclotriphosphazene (HPCTP)	15	51	513	82	2.85	—	V-0	[128]
		63	1321	157	—	—	NR	[129]
Hexaphenoxycyclotriphosphazene (HPCTP)	15	54	513	82	4.22	—	V-0	[129]
		100	733	141	—	21	HB	[130]
Tetraphenylphosphonium modified montmorillonite (TPP-MMT)	5	110	482	140	1.68	25	HB	[130]
		47	891	151	—	21	HB	[130]
Tetraphenylphosphonium modified montmorillonite (TPP-MMT)	5	53	571	138	1.92	25	HB	[130]
		22	1196	147	—	21	HB	[130]
Tetraphenylphosphonium modified montmorillonite (TPP-MMT)	5	25	694	140	2.05	25	HB	[130]
		49	904	95	—	21	NR	[131]
hyperbranched poly(phosphoester) (*hb*PPE)	10	49	506	62	2.73	23.6	HB	[131]
hyperbranched poly(phosphoester) (*hb*PPE)	20	49	699	53	2.31	25.9	HB	[131]
	0	58	1126.3	100.36	—	26.1	—	[132]
poly(cyclotriphosphazeneco-4,4′-sulfonyldiphenol) (PZS)	3	61	986.5	91.89	1.31	28.6	—	[132]
hybrid poly(cyclotriphosphazeneco-4,4′-sulfonyldiphenol)-strontium hydroxystannate nanorod (PZS@SrSn(OH)_6_)	3	60	801.2	88.96	1.64	29.5	—	[132]
	0	36.6	970.9	59.1	—	19.8	—	[133]
1-oxo-4-hydroxymethyl-2,6,7-trioxa-l-phosphabicyclo [2.2.2] octane modified trimellitic anhydride chloride (PEPA-TMAC)	16.5	30.1	523.7	42	2.14	23.4	—	[133]
1-oxo-4-hydroxymethyl-2,6,7-trioxa-l-phosphabicyclo [2.2.2] octane modified trimellitic anhydride chloride (PEPA-TMAC)	33	33.9	337.2	36.9	4.27	26.9	—	[133]
		50	986	91.1	—	25.9	—	[134]
poly(cyclotriphosphazene-c-sulfonyldiphenol) (PCPS)	1	49	979	92.1	0.97	27	—	[134]
poly(cyclotriphosphazene-c-sulfonyldiphenol) (PCPS)	3	44	500	85.8	1.84	29.8	—	[134]
poly(cyclotriphosphazene-c-sulfonyldiphenol) (PCPS)	5	43	542	78.7	1.81	30.5	—	[134]
		60	1146	56	—	26.5	—	[135]
Boron phosphate: reaction between boric acid & phosphoric acid by calcining at 300 ˚C (BP1)	5	53	652	31	2.80	29.6	—	[135]
Boron phosphate: reaction between boric acid & phosphoric acid by calcining at 400 ˚C (BP2)	5	53	654	34	2.54	29.7	—	[135]
Boron phosphate: reaction between boric acid & phosphoric acid by calcining at 500 ˚C (BP3)	5	54	681	33	2.57	29.6	—	[135]
Boron phosphate: reaction between boric acid & phosphoric acid by calcining at 600 ˚C (BP4)	5	56	710	38	2.22	29.3	—	[135]
Boron phosphate: reaction between boric acid & phosphoric acid by calcining at 700 ˚C (BP5)	5	56	754	38	2.09	29	—	[135]
		86	1650	213	—	20.2	—	[136]
3-((Methoxydiphenylsilyl) oxy)-9-methyl-2, 4, 8, 10-tetraoxa-3, 9-diphosphaspiro [5. 5] undecane 3, 9-dioxide (SDPS)	10.4	62	1378	203	0.90	28.9	—	[136]
		48	1023	109	—	22.2	—	[137]
dibenzylphosphinic acid modified aluminum hydroxide (AOPH-NR)	4.25	79	789	101	2.30	28	—	[137]
diallylphosphinic acid modified aluminum hydroxide (AOPH-C1)	4.25	80	1092	107	1.59	23.4	—	[137]
bis(3-methoxy-3-oxopropyl)phosphinic acid modified aluminum hydroxide (AOPH-C2)	4.25	58	1063	99	1.28	23.6	—	[137]
bis(2-cyanoethyl)phosphinic acid modified aluminum hydroxide (AOPH-C3)	4.25	78	1024	106	1.66	23.8	—	[137]
epoxy acrylate		41	889	28.3	—	21	—	[138]
N,N-bis(2-hydroxyethyl acrylate) aminomethyl phosphonic acid diethylester (BHAAPE)	5	35	719	25.3	1.18	28	—	[138]
N,N-bis(2-hydroxyethyl acrylate) aminomethyl phosphonic acid diethylester (BHAAPE)	10	25	590	23.7	1.09	30	—	[138]
N,N-bis(2-hydroxyethyl acrylate) aminomethyl phosphonic acid diethylester (BHAAPE)	20	19	508	22.3	1.02	31	—	[138]
	0	25	1113	222.9	—	—	—	[139]
ammonium polyphosphate (APP)	10	35	685.9	127.4	3.97	—	—	[139]
	0	60	2187	124	—	—	—	[140]
poly (cyclotriphosphazene-co-4,4′-sulfonyldiphenol) (PZS)	2	57	1871	101	1.36	—	—	[140]
poly (cyclotriphosphazene-co-4,4′-sulfonyldiphenol)@molybdenum disulfide nanoflower (PZS@MoS_2_)	2	52	1335	91	1.93	—	—	[140]
poly (cyclotriphosphazene-co-4,4′-sulfonyldiphenol)@molybdenum disulfide nanoflower (PZS@MoS_2_)	3	56	1251	85	2.38	—	—	[140]
	0	19	980	81	—	—	—	[141]
N,N′-dibutyl-phosphate diamide assembled into the cavity of β-cyclodextrin (DBPDA-βCD)	3	19	756	75	1.40	—	—	[141]
	0	78	2116	167.1	—	—	—	[142]
Polyphosphazene functionalized black phosphorus nanosheets (BP-PZN)	0.5	78	1613.7	119.8	1.82	—	—	[142]
Polyphosphazene functionalized black phosphorus nanosheets (BP-PZN)	1	85	1082.1	73.5	4.84	—	—	[142]
Polyphosphazene functionalized black phosphorus nanosheets (BP-PZN)	2	81	859.5	60.8	7.02	—	—	[142]
black phosphorus bulk nanosheets (BP-Bulk)	2	87	1082.3	94.3	3.86	—	—	[142]
		63	1396.9	81.3	—	—	—	[143]
ene-terminated hyperbranched polyphosphate acrylate (HPPA)	2	57	1096.9	75.4	1.24	—	—	[143]
ene-terminated hyperbranched polyphosphate acrylate-thiol-functionalized mesoporous silica (HPPA-SH-mSiO_2_)	2	62	995.3	68.3	1.64	—	—	[143]
		76	850	88	—	—	—	[144]
phosphorous metal-organic framework (P-MOF)	0.5	75	766	84	1.14	—	—	[144]
phosphorous metal-organic framework (P-MOF)	1	79	728	71	1.50	—	—	[144]
phosphorous metal-organic framework (P-MOF)	2	70	615	69	1.62	—	—	[144]
		53	1484	86.3	—	—	—	[145]
cardanol-derived zirconium phosphate (CZrP)	2	56	1122	76.1	1.58	—	—	[145]
cardanol-derived zirconium phosphate (CZrP)	4	50	970	73.2	1.70	—	—	[145]
cardanol-derived zirconium phosphate (CZrP)	6	54	858	67.8	2.24	—	—	[145]
zirconium phosphate (ZrP)	6	51	1248	85.5	1.15	—	—	[145]
		24	1002.4	104.1	—	—	—	[146]
Dimethyl methylphosphonate loaded halloysite nanotube (DMMP-HNT)	20	24	578.1	73.8	2.44	—	—	[146]
		54	1068	76	—	21	—	[147]
melamine poly(magnesium phosphate) (S600)	20	44	298	57	3.89	—	—	[147]
aluminium diethylphosphinate (AlPi)	20	41	492	56	2.23	—	—	[147]
melamine polyphosphate (MPP)	20	38	244	26	9.00	—	—	[147]
		74	1915.3	107.6	—	—	—	[148]
poly-(cyclotriphos pazene-co-4,40-diaminodiphenyl ether) surface modified silica nanospheres (SiO_2_@PZM)	1	80	1363.4	86.8	1.88	—	—	[148]
poly-(cyclotriphos pazene-co-4,40-diaminodiphenyl ether) surface modified silica nanospheres-cuprous (SiO_2_@PZM@Cu)	1	74	1289.3	78	2.04	—	—	[148]
poly-(cyclotriphos pazene-co-4,40-diaminodiphenyl ether) surface modified silica nanospheres-cuprous (SiO_2_@PZM@Cu)	2	80	1188.8	73.9	2.53	—	—	[148]
		82	1820.7	99.3	—	—	—	[149]
functionalized polyphosphazene nanotubes wrapped with a cross-linked DOPO-based flame retardant (FR@PZS)	0.5	82	1584.2	87	1.31	—	—	[149]
functionalized polyphosphazene nanotubes wrapped with a cross-linked DOPO-based flame retardant (FR@PZS)	1	82	1298.2	80.8	1.72	—	—	[149]
functionalized polyphosphazene nanotubes wrapped with a cross-linked DOPO-based flame retardant (FR@PZS)	3	82	982.6	72.4	2.54	—	—	[149]
polyphosphazene nanotube (PZS)	3	82	1152.5	83.9	1.86	—	—	[149]
		38	943	60.3	—	—	—	[150]
ammonium polyphosphate (APP)	5	36	543	58.8	1.68	—	—	[150]
		45	855	118	—	—	—	[151]
polyhedral oligomeric silsesquioxane containing 9,10-dihydro-9-oxa-10-phosphaphenanthrene-10-oxide (DOPO-POSS)	20	57	431	91	3.25	—	—	[151]
Epoxy acrylic		32	223.4	30.8	—	—	—	[152]
ammonium polyphosphate (APP)	30	35	225.2	30.7	1.08	—	—	[152]
Co-microencapsulated ammonium polyphosphate and pentaerythritol (M(APP & PER))	30	58	233.2	27.3	1.95	—	—	[152]
		29	2467	164	—	—	—	[153]
Triphenylphosphite (TPPi)	15	21	504	114	5.09	—	—	[153]
Triphenylphosphate (TPPa)	15	12	1959	128	0.66	—	—	[153]
triphenylphosphine oxide (TPPO)	15	34	1310	126	2.87	—	—	[153]
	—	32	2572	184	—	—	—	[154]
poly(m-phenylene methyl 1phosphonate) (PMP)	11.4	12	724	102	2.40	—	—	[154]
9,10-dihydro-9-oxa-10phosphaphenanthrene-10-oxide (DOPO)	13.9	7	1286	100	0.80	—	—	[154]
red phosphorus (RP)	4.3	7	1614	156	0.41	—	—	[154]
aluminum diethylphosphinate (OP)	8.3	7	1480	146	0.47	—	—	[154]
		33	910	97.54	—	—	—	[155]
IFR: reaction between phosphorus acid & melamine & pentaerythritol with the molar ratio of 1:1:2.12 (IFR)	30	38	357	80.35	3.56	—	—	[155]
IFR: reaction between phosphorus acid & melamine & pentaerythritol with the molar ratio of 1:1:2.12 (IFR)	30	65	350	82.28	6.07	—	—	[155]
IFR: reaction between phosphorus acid & melamine & pentaerythritol with the molar ratio of 1:1:2.12 (IFR)	30	71	263	73.25	9.91	—	—	[155]
		67	979.7	128	—	—	—	[156]
Butyl phosphate ester (EPE)	33.3	35	203.3	87	3.70	—	—	[156]
Ethylphosphonate ester (EPE)	33.3	76	304.8	80	5.83	—	—	[156]
Butanediol and butanol mixed phosphate ester (BBPE)	33.3	76	300.4	83	5.70	—	—	[156]
Butanediol and octanol mixed phosphate ester (BOPE)	33.3	79	296.9	91	5.47	—	—	[156]
Hexanediol and butanol mixed phosphate ester (HBPE)	33.3	82	283.1	88	6.16	—	—	[156]
		32	910	98	—	—	—	[157]
IFR: reaction between phosphorus acid & melamine & pentaerythritol with the molar ratio of 1:1:2 (IFR)	30	61	341	68	7.33	—	—	[157]
IFR: reaction between phosphorus acid & melamine & pentaerythritol with the molar ratio of 1:1:2 (IFR)	30	41	248	73	6.31	—	—	[157]
IFR: reaction between phosphorus acid & melamine & pentaerythritol with the molar ratio of 1:1:2 (IFR)	30	41	268	68	6.26	—	—	[157]
IFR: reaction between phosphorus acid & melamine & pentaerythritol with the molar ratio of 1:1:2 (IFR)	30	45	237	71	7.45	—	—	[157]
		94	1097.2	119	—	—	—	[158]
phosphorus oxychloride & pentaerythritol (POCl3 & PER) modified expandable graphite (EGM)	5	76	276.2	136	2.81	—	—	[158]
phosphorus oxychloride & pentaerythritol (POCl3 & PER) modified expandable graphite (EGM)	15	45	184.1	88	3.85	—	—	[158]
		54	1327	99.1	—	—	—	[159]
Phosphorylated chitosan modified montmorillonite intercalation iron compounds (PCTS-Fe-OMMT)	1	51	1071	88.3	1.31	—	—	[159]
Phosphorylated chitosan modified montmorillonite intercalation iron compounds (PCTS-Fe-OMMT)	3	48	917	86.8	1.46	—	—	[159]
Phosphorylated chitosan modified montmorillonite intercalation iron compounds (PCTS-Fe-OMMT)	5	44	794	82.2	1.64	—	—	[159]
		41	1222	159	—	—	—	[160]
ammonium polyphosphate (APP)	20	49	879	105	2.51	—	—	[160]
ammonium polyphosphate (APP)	40	56	225	55	21.44	—	—	[160]
	0	47	1630	82.3	—	—	—	[161]
9,10-dihydro-9-oxa-10-phosphaphenanthrene-10-oxide-phosphonamidate functionalized reduced graphene oxide(DOPOph-RGNO)	1	49	1268	62.3	1.77	—	—	[161]
9,10-dihydro-9-oxa-10-phosphaphenanthrene-10-oxide-phosphonamidate functionalized reduced graphene oxide(DOPOph-RGNO)	2	43	1248	55	1.78	—	—	[161]
9,10-dihydro-9-oxa-10-phosphaphenanthrene-10-oxide-phosphonamidate functionalized reduced graphene oxide(DOPOph-RGNO)	3	45	1117	54	2.12	—	—	[161]
	0	21	453.5	36.2	—	22.1	NR	[120]
melamine coated ammonium polyphosphate (Mel-APP) ^a^	9.59	20	290.4	32.2	1.67	32	V-1	[120]
	0	53	387	24.3	—	31	NR	[24]
N, N′-diallyl-p-phenylphosphonicdiamide (FP1) ^b^	2.6	49	423	20.4	1.00	43	NR	[24]
	0	54	508.3	47.8	—	31	NR	[162]
polyelectrolyte complexes consisting of chitosan & ammonium polyphosphate (PEC) ^c^	5.2	51	358	44	1.45	36	NR	[162]
polyelectrolyte complexes consisting of chitosan & ammonium polyphosphate (PEC) ^c^	6.9	50	307.5	39.6	1.84	38.5	V-1	[162]
polyelectrolyte complexes consisting of chitosan & ammonium polyphosphate (PEC) ^c^	8.1	49	255.9	35.5	2.42	40.5	V-0	[162]
		51	347	26.2	—	33.2	HB	[113]
9,10-dihydro-9-oxy-10-phosphaphenanthrene-10-oxide units linked to the star-shaped aliphatic ground body tetra-[(acryloyloxy)ethyl] pentarythrit (DOPP) ^d^	5.9	56	248	19.9	2.02	45.3	V-0	[113]
9,10-dihydro-9-oxy-10-phosphaphenanthrene-10-oxide units linked to the star-shaped aliphatic ground body heterocyclic tris-[(acryloyloxy)ethyl] isocyanurate (DOPI) ^d^	6.9	60	247	20	2.16	47.7	V-0	[113]
		24	451	37	—	—	NR	[126]
Melamine coated ammonium polyphosphate (Mel-APP) ^e^	14.6	22	233	11	5.96	—	V-1	[126]
	—	42	385	21.8	—	27.5	—	[163,164]
IFR: contains melamine phosphate (IFR) ^f^	4.7	35	278	18.3	1.37	35.2	—	[163,164]
		28	349	20.4	—	—	—	[150]
ammonium polyphosphate (APP) ^g^	5	24	345	18.6	0.95	—	—	[150]
		21.2	720.5	68	—	—	—	[165]
ammonium polyphosphate (APP) ^h^	3.15	20.3	375.3	42	2.97	—	—	[165]
ammonium polyphosphate (APP) ^h^	8.88	18.1	293.8	33	4.31	—	—	[165]
ammonium polyphosphate (APP) ^h^	16.32	21	186.7	27	9.62	—	—	[165]
		44	853	51.9	—	—	—	[166]
melamine phosphate (MP) ^i^	5	38	528	48.8	1.48	—	—	[166]
9,10-Dihydro-9-oxa-10-phosphaphenanthrene-10-oxide (DOPO) ^i^	5	34	624	41.3	1.32	—	—	[166]
	0	39	456	38	—	—	—	[167]
IFR: contains melamine phosphate (IFR) ^j^	5	35	374	28.8	1.44	—	—	[167]
IFR: contains melamine phosphate (IFR) ^j^	10	50	226	17.3	5.68	—	—	[167]
IFR: contains melamine phosphate (IFR) ^j^	15	94	253	18.6	8.87	—	—	[167]
		55	754	61.3	—	—	—	[168]
ammonium polyphosphate (APP) ^k^	15	46	259	34.4	4.33	—	—	[168]
		39	642	64.2	—	—	—	[168]
ammonium polyphosphate (APP) ^l^	15	44	232	40.1	4.99	—	—	[168]

^a^ Matrix: eight layers of Woven E-glass fabric reinforced epoxy; ^b^ Matrix: six layers of dry carbon fiber fabric reinforced RTM6 epoxy; ^c^ Matrix: Unidirectional carbon fiber reinforced epoxy resin; ^d^ Matrix: Carbon fibers reinforced epoxy; ^e^ Matrix: eight layers of Woven E-glass fabric reinforced epoxy; ^f^ Matrix: eight layers of woven E-glass reinforced film of multifunctional epoxy resin; ^g^ Matrix: carbon fiber reinforced epoxy resin; ^h^ Matrix: four fabric layers of unidirectional hemp fabric reinforced epoxy; ^I^ Matrix: eight layers of woven roving glass fabric reinforced epoxy phenol novolak resin blend; ^j^ Matrix: eight layers of woven E-glass reinforced epoxy; ^k^ Matrix: six layers of plain weave hemp fabric-reinforced epoxy; ^l^ Matrix: six layers of plain weave Hemp fabrics treated with water glass-reinforced epoxy.

**Table 2:** The state of flame retardancy performance of epoxy resins containing nonphosphorus flame retardants in terms of FRI (* the name and percentage of incorporated flame retardant is given after each epoxy resin). The notes *a* to *h* on the bottom of the table are representative of composite systems containing woven or nonwoven fibers.

Epoxy Resins and Incorporated Non Phosphorus FR *	wt.%	TTI (s)	pHRR (kW.m^−^^2^)	THR (MJ·m^−^^2^)	FRI	LOI	UL94	Ref.
	0	11	781	142	—	21.8		[169]
(2,4,6-tris(4-boronic-2-thiophene)-1,3,5-triazine (3TT-3BA)	20	17	454	108	3.49	31.2	V-0	[169]
	0	32	827	116	—	21.8	NR	[28]
graphene nanosheet (GN)	3	35	560	113	1.65	26.7	NR	[28]
	0	32	781	107	—	21.8	NR	[29]
multiwalled carbon nanotube (MWCNT)	0.8	40	473	97	2.27	21.2	NR	[29]
	0	32	781	107	—	21.8	NR	[30]
Organically modified montmorillonite (DK4:two longchain alkyl ammonium modified montmorillonite) (OMMT)	7	40	576	98	1.85	23.7	NR	[30]
	0	32	781	107	—	21.8	NR	[31]
organomodified magnesium aluminium layered double hydroxide (OLDH)	1	35	543	121	1.39	21.9	NR	[31]
organomodified magnesium aluminium layered double hydroxide (OLDH)	5	35	521	104	1.68	23.6	V-0	[31]
organomodified magnesium aluminium layered double hydroxide (OLDH)	10	49	391	106	3.08	22.1	V-0	[31]
	0	71	1146	56	—	21.2	NR	[170]
magnesium aluminium layered double hydroxide (MgAl-LDH)	2	63	865	49	1.34	23.8	NR	[170]
zeolitic imidazolate framework8 (ZIF8)	2	58	886	41	1.44	23.3	NR	[170]
zeolitic imidazolate framework8 decorated magnesium aluminium layered double hydroxide (ZIF8@MgAl-LDH)	2	54	562	39	2.22	24.7	V-1	[170]
zeolitic imidazolate framework67 (ZIF67)	2	62	817	42	1.63	23.6	NR	[170]
zeolitic imidazolate framework67 decorated MgAl-layered double hydroxide (ZIF67@MgAl-LDH)	2	56	432	34	3.44	25.5	V-1	[170]
	0	61	1208	77.3	—	22.5	NR	[52]
triazine-based flame retardant (TAT)	20	42	1030	75.8	0.82	24.1	NR	[52]
		35	1065	80.3	—	22.9	NR	[171]
2,4,6-tris-(4-boronphenoxy)-(1,3,5)-triazine (TNB)	1	23	686	68.1	1.20	26.1	V-1	[171]
2,4,6-tris-(4-boronphenoxy)-(1,3,5)-triazine (TNB)	5	22	427	64.1	1.96	28.3	V-1	[171]
2,4,6-tris-(4-boronphenoxy)-(1,3,5)-triazine (TNB)	10	20	324	59.3	2.54	29.4	V-1	[171]
2,4,6-tris-(4-boronphenoxy)-(1,3,5)-triazine (TNB)	15	22	309	58.3	2.98	30.4	V-0	[171]
2,4,6-tris-(4-boronphenoxy)-(1,3,5)-triazine (TNB)	20	22	305	58	3.03	31.2	V-0	[171]
		53	1121	102	—	20	NR	[55]
Cuprous oxide (Cu_2_O)	21	47	1007	86	1.17	22	NR	[55]
		45	1091	83	—	22.8	NR	[56]
magnesium hydroxide (MH)	3	38	751	80	1.27	25.2	NR	[56]
		60	873	88.5	—	22.5	NR	[63]
2,4,6-triphenoxy-1,3,5-triazine (TN)	3.42	25	943	78.4	0.43	29	NR	[63]
		58	1208	80.6	—	22.5	NR	[66]
expandable graphite (EG)	20	49	225	63.3	5.77	31	NR	[66]
		57	1557	94.5	—	24.5	NR	[67]
nucleophilic substitution reaction between N-(4-hydroxyphenyl) maleimide & cyanuric chloride (TMT)	8	52	1395	88.4	1.08	27	NR	[67]
		61	1208	80.6	—	22.5	NR	[68]
nucleophilic substitution reaction between N-(4-hydroxyphenyl) maleimide & cyanuric chloride (TMT)	7	61	858	73.5	1.54	25.5	NR	[68]
		56	1420	140	—	26	NR	[77]
organically modified montmorillonite (OMMT)	1	39	1540	116	0.77	29.3	NR	[77]
		69	966	93.9	—	22.5	NR	[78]
triallyl isocyanurate (TAIC)	10	61	1306	123	0.50	23.6	NR	[78]
		52	995	93.3	—	22.5	NR	[81]
Triphenoxy-1,3,5-triazine (TPT)	14	48	964	88.7	1.00	24.5	NR	[81]
		67	950	98	—	24.1	NR	[172]
Halloysite nanotube (HNT)	5	65	1170	93	0.83	26.1	NR	[172]
Halloysite nanotube (HNT)	10	65	1002	95	0.94	25.4	NR	[172]
biomimetic polydopamine nanocoating functionalized Halloysite nanotube (HNT@PDA)	5	65	1088	104	0.79	25.6	NR	[172]
biomimetic polydopamine nanocoating functionalized Halloysite nanotube (HNT@PDA)	10	67	881	91	1.16	25.6	NR	[172]
biomimetic polydopamine nanocoating functionalized Halloysite nanotube and ultrafine Fe(OH)_3_ nanoparticles (HNT@PDA@Fe(OH)_3_)	5	61	695	90	1.35	33.9	V-1	[172]
biomimetic polydopamine nanocoating functionalized Halloysite nanotube and ultrafine Fe(OH)_3_ nanoparticles (HNT@PDA@Fe(OH)_3_)	10	58	698	88	1.31	33.8	NR	[172]
		50	860	133	—	23	NR	[94]
Montmorillonite (MMT)	6	49	792	100	1.41	26	NR	[94]
		45	855	112	—	25	NR	[102]
octaphenyl polyhedral oligomeric silsesquioxane (OPS)	5	60	712	103	1.74	31.1	NR	[102]
		45	855	112	—	25	NR	[103]
Octaphenyl silsesquioxane (OPS)	4.1	55	626	112	1.66	27.2	NR	[103]
Polyphenyl silsesquioxane (PPSQ)	4.1	50	925	116	0.99	27.1	NR	[103]
		45	855	112	—	25	NR	[104]
Octaphenyl silsesquioxane (OPS)	4.1	55	626	112	1.66	27.2	NR	[104]
Octaaminophenylsilsesquioxane (OAPS)	4.6	57	635	110	1.73	27	NR	[104]
		50	860	112	—	23	NR	[106]
Octaphenyl polyhedral oligomeric silsesquioxane (OPS)	4.1	55	626	112	1.51	25	NR	[106]
		57	459	55.2	—	19.5	NR	[173]
aluminum trihydroxide (ATH)	40	68	231	41.2	3.17	23.6	NR	[173]
Colemanite (C)	40	58	158	34.3	4.75	23.6	NR	[173]
Ulexite (U)	40	62	171	38.2	4.21	22.6	NR	[173]
boric acid (BA)	40	76	132	32.1	7.97	28.5	V-0	[173]
boric oxide (BO)	40	68	82	20.6	17.89	24.2	NR	[173]
melamine borate (MB)	30	78	107	26.9	12.05	24.5	V-0	[173]
guanidinium nonaborate (GB)	30	65	105	26.8	10.27	23.6	NR	[173]
		64	821	94	—	23.2	NR	[115]
polyhedral oligomeric octadiphenylsulfonylsilsesquioxane (ODPSS)	5	59	417	74	2.30	24.3	NR	[115]
		60	920	90.5	—	22.7	NR	[116]
Magnesium-Aluminum layered double hydroxide (Mg-Al LDH)	4	53	835	89.6	0.98	24.3	NR	[116]
		108	1634	78	—	19.8	NR	[174]
Trisilanolisobutyl Polyhedral oligomeric silsesquioxane (T8POSS)	10	99	774	56	2.69	20.7	NR	[174]
triglycidyl isocyanurate (TGIC)	10	86	1190	67	1.27	19.9	NR	[174]
	0	51	1914	81.9	—	22	NR	[121]
reduced graphene oxide (RGO)	1	47	1356	67.6	1.57	23.5	NR	[121]
		21	1910	84.4	—	22.1	NR	[120]
halloysite nano-tube (HNT)	2	20	1591	90.7	1.06	19.5	NR	[120]
layered double hydroxide (LDH)	2	21	803	87.5	2.29	21.6	NR	[120]
layered double hydroxide (LDH)	4	22	861	85.4	2.29	20. 6	NR	[120]
layered double hydroxide (LDH)	6	20	791	82.9	2.34	19.7	NR	[120]
epoxy novolac resin	0	51	682	110	—	—	NR	[124]
Boehmite (AlO(OH))	30	69	535	88	2.15	—	V-1	[124]
	0	50	992	91	—	—	NR	[125]
activated carbon spheres (ACS)	2	56	898	91	1.23	—	—	[125]
activated carbon spheres@SnO_2_ hybrid (ACS@SnO_2_)	2	50	761	98	1.21	—	—	[125]
activated carbon spheres@SnO_2_@NiO hybrid (ACS@SnO_2_@NiO)	2	56	839	92	1.31	—	NR	[125]
	0	50	986	91	—	—	NR	[125]
activated carbon spheres@SnO_2_@NiO hybrid (ACS@SnO_2_@NiO)	5	51	823	88	1.26	—	NR	[125]
		63	1321	157	—	—	NR	[129]
octapropylglycidylether polyhedral oligomeric silsesquioxane (OGPOSS)	15	60	1026	145	1.32	—	NR	[129]
	0	19	1325	95.7	—	19.2	HB	[33]
Expandable graphite (EG)	15	34	1015	85.3	2.61	25.4	HB	[33]
		100	733	141	—	21	HB	[130]
Silicate glass (CP)	10	101	315	139	2.38	25	HB	[130]
Silicate glass (CP)	15	89	268	132	2.60	24	HB	[130]
		47	891	151	—	21	HB	[130]
Silicate glass (CP)	10	44	408	136	2.27	25	HB	[130]
Silicate glass (CP)	15	46	346	134	2.84	24	HB	[130]
		22	1196	147	—	21	HB	[130]
Silicate glass (CP)	10	20	565	137	2.06	25	HB	[130]
Silicate glass (CP)	15	19	585	129	2.01	24	HB	[130]
	0	58	1126	100	—	26.1	—	[132]
strontium hydroxystannate nanorod (SrSn(OH)_6_)	3	55	889	92.6	1.30	28.4	—	[132]
	0	73	1054	39.1	—	22.4	—	[175]
silica nanoparticles (SiO_2_)	2	65	727	34.4	1.46	26	—	[175]
Zeolitic imidazolate framework-8 nanocrystals (ZIF8)	2	60	431	25.3	3.10	26.9	—	[175]
Zeolitic imidazolate framework-8 coated with SiO_2_ (ZIF8@SiO_2_)	2	68	254	23.9	6.32	28.1	—	[175]
	0	69	1150	54.7	—	22	—	[176]
molybdenum disulfide (MoS_2_)	2	65	854	41.7	1.66	25.7	—	[176]
titanium dioxide nanotube (TNT)	2	58	815	39.5	1.64	25.5	—	[176]
molybdenum disulfide decorated titanium dioxide nanotube (MoS_2_-TNT)	1	63	859	43.7	1.53	25.1	—	[176]
molybdenum disulfide decorated titanium dioxide nanotube (MoS_2_-TNT)	2	60	701	37.1	2.10	26.8	—	[176]
molybdenum disulfide decorated titanium dioxide nanotube (MoS_2_-TNT)	3	61	627	32.1	2.76	28.1	—	[176]
		45	1193	76	—	23.8	—	[177]
Sepiolite (Sep)	2	49	1288	78	0.98	29.8	—	[177]
Sepiolite (Sep)	4	61	963	101	1.26	30.1	—	[177]
Fe3O4-doped sepiolite (Fe_3_o_4_–Sep)	2	42	1093	83	0.93	33.8	—	[177]
Fe3O4-doped sepiolite (Fe_3_o_4_–Sep)	4	45	883	89	1.15	36.7	—	[177]
		45	1193	76	—	23.8	—	[178]
oxidized graphene nanoplatelets (GNO)	1	49	1204	81	1.01	25.2	—	[178]
oxidized graphene nanoplatelets (GNO)	3	47	1244	72	1.05	25.6	—	[178]
Cu-doped graphene (GN-Cu)	1	45	825	66	1.66	25.8	—	[178]
Cu-doped graphene (GN-Cu)	3	47	786	64	1.88	26.4	—	[178]
	0	54	1068	76	—	21	—	[147]
Boehmite (AlO(OH))	20	49	870	65	1.30	—	—	[147]
		54	1068	75.8	—	—	HB	[127]
Boehmite (AlO(OH))	20	49	870	65.5	1.28	—	HB	[127]
amorphous silicon dioxide (SiO_2_)	20	41	907	57.6	1.17	—	HB	[127]
Bisphenol-A	0	22	1680	79	—	—	—	[179]
α-Manganese dioxide nanosheets (α-MnO_2_)	0.5	25	1701	77	1.15	—	—	[179]
α-Manganese dioxide nanosheets (α-MnO_2_)	1	24	1480	73	1.34	—	—	[179]
α-Manganese dioxide nanosheets (α-MnO_2_)	2	23	1400	67	1.47	—	—	[179]
δ-Manganese dioxide nanosheets (δ-MnO_2_)	0.5	25	1617	74	1.26	—	—	[179]
δ-Manganese dioxide nanosheets (δ-MnO_2_)	1	26	1547	74	1.37	—	—	[179]
δ-Manganese dioxide nanosheets (δ-MnO_2_)	2	27	1358	64	1.87	—	—	[179]
	0	60	2187	124	—	—	—	[140]
molybdenum disulfide nanoflower (MoS_2_)	2	49	1457	98	1.55	—	—	[140]
	0	47.7	1308	86.8	—	—	—	[180]
Aminopropylisobutyl polyhedral oligomeric silsesquioxane (AI-POSS)	7.2	44.3	880	83.6	1.43	—	—	[180]
Aminopropylisobutyl polyhedral oligomeric silsesquioxane (AI-POSS)	21.8	36.3	585	97.7	1.51	—	—	[180]
Aminopropylisobutyl polyhedral oligomeric silsesquioxane (AI-POSS)	54	32.2	616	65.3	1.90	—	—	[180]
	0	5	986	113	—	—	—	[181]
Expandable graphite (EG)	9	10	152	110	13.33	—	—	[181]
halloysite nanotube (HNT)	9	5	969	110	1.04	—	—	[181]
	0	117	1184	95.3	—	—	—	[182]
Boron Nitride with D50 = 2 μm (BN 2 μm)	45	175	767	71.5	3.07	—	—	[182]
Boehmite with D50 = 2 μm (BT 2 μm)	45	140	674	72.2	2.77	—	—	[182]
	0	22	1650	80	—	—	—	[183]
Manganese dioxide (MnO_2)_	2	27	1443	71	1.58	—	—	[183]
Manganese dioxide@zinc hydroxystannate binary hybrid (MnO_2_@ZHS)	0.5	24	1487	56	1.72	—	—	[183]
Manganese dioxide@zinc hydroxystannate binary hybrid (MnO_2_@ZHS)	1	25	1275	49	2.40	—	—	[183]
Manganese dioxide@zinc hydroxystannate binary hybrid (MnO_2_@ZHS)	2	23	989	61	2.28	—	—	[183]
Diglycidyl ether of bisphenol-F epoxy	0	66	1197	82.7	—	—	—	[184]
ionic liquid flame retardant (ILFR)	5	55	753	62.5	1.75	—	—	[184]
boron nitride nanosheets (BN)	5	70	813	68.2	1.89	—	—	[184]
ionic liquid flame retardant functionalized boron nitride nanosheets (ILFR-fBN)	5	104	689	51.5	4.39	—	—	[184]
		63	1397	81.3	—	—	—	[143]
thiol-functionalized mesoporous silica (SH-mSiO_2_)	2	65	1117	77.8	1.34	—	—	[143]
		52	972	99	—	—	—	[185]
short carbon fiber (SCF)	0.5	69	793	92	1.75	—	—	[185]
short carbon fiber (SCF)	0.7	80	723	88	2.32	—	—	[185]
short carbon fiber (SCF)	1	62	840	89	1.53	—	—	[185]
short carbon fiber (SCF)	1.5	98	793	101	2.26	—	—	[185]
		24	1002	104	—	—	—	[146]
halloysite nanotube (HNT)	20	43	790	75.2	3.14	—	—	[146]
		38	1542	76.2	—	—	—	[186]
nanomer I.28E organoclay (m-Clay)	2.5	58	1298	56.6	2.44	—	—	[186]
Deoxyribonucleic Acid modified clay (d-Clay)	2.5	55	1220	52.4	2.66	—	—	[186]
		22	1032	49.2	—	—	—	[187]
Layered double hydroxide (LDH)	3	27	968	49.6	1.29	—	—	[187]
β-Iron oxyhydroxide (β-FeOOH)	3	25	857	48	1.40	—	—	[187]
Layered double hydroxide nanosheet-wrapped β-Iron oxyhydroxide rod hybrid (LDH-β-FeOOH)	3	20	736	44.8	1.40	—	—	[187]
		47	1083	45.7	—	—	—	[188]
amorphous hydrous TiO_2_ solid spheres (AHTSS)	0.5	52	1125	46	1.05	—	—	[188]
amorphous hydrous TiO_2_ solid spheres (AHTSS)	2	53	951	43.6	1.34	—	—	[188]
urchin-like mesoporous TiO_2_ hollow spheres (UMTHS)	0.5	52	827	43.3	1.52	—	—	[188]
urchin-like mesoporous TiO_2_ hollow spheres (UMTHS-2)	2	52	706	38.5	2.01	—	—	[188]
		65	1592	39.7	—	—	—	[189]
chitosan-modified molybdenum disulfide nanosheets (CS-MoS_2_)	0.5	71	1243	35.9	1.54	—	—	[189]
chitosan-modified molybdenum disulfide nanosheets (CS-MoS_2_)	1	74	1107	28.6	2.27	—	—	[189]
chitosan-modified molybdenum disulfide nanosheets (CS-MoS_2_)	2	75	902	33.9	2.38	—	—	[189]
molybdenum disulfide nanosheets (MoS_2_)	2	72	1178	40.1	1.48	—	—	[189]
		74	1915	108	—	—	—	[148]
silica nanospheres (SiO_2_)	1	74	1777	95.6	1.21	—	—	[148]
		38	943	60.3	—	—	—	[150]
carbon nanotube (CNT)	1	26	673	53.8	1.07	—	—	[150]
chemical treatment carbon nanotube (CCNT)	1	32	837	57.4	0.99	—	—	[150]
thermal treatment carbon nanotube (TCNT)	1	25	585	56.6	1.13	—	—	[150]
layered double hydroxide (LDH)	5	35	578	58.4	1.55	—	—	[150]
Hydrogenated fatty acid modified layered double hydroxide (OLDH)	5	38	453	66.5	1.88	—	—	[150]
Montmorillonite (MMT)	5	38	717	58.6	1.35	—	—	[150]
Quaternary ammonium salt modified montmorillonite (OMMT)	5	33	823	61.7	0.97	—	—	[150]
aluminium trihydroxide (ATH)	5	35	617	59.2	1.43	—	—	[150]
		65	993	141	—	—	—	[190]
Expanded graphite (EG)	5	68	1188	125	0.98	—	—	[190]
Expanded graphite (EG)	10	80	1487	113	1.02	—	—	[190]
Expanded graphite (EG)	15	102	1911	124	0.92	—	—	[190]
Expanded graphite (EG)	23	116	1992	102	1.23	—	—	[190]
Expanded graphite (EG)	50	132	1800	81	1.95	—	—	[190]
		141	932	74.3	—	—	—	[191,192]
Bentonite (BT)	3	150	1094	74	0.91	—	—	[191,192]
Bentonite (BT)	5	158	1192	88.1	0.73	—	—	[191,192]
6-(4-butylphenyl)21,3,5-triazine-2,4-diamine modified bentonite (BFTDA-BT)	3	140	966	74.1	0.96	—	—	[191,192]
6-(4-butylphenyl)21,3,5-triazine-2,4-diamine modified bentonite (BFTDA-BT)	5	145	998	82.2	0.86	—	—	[191,192]
11-amino-N-(pyridine-2yl)undecanamide modified bentonite (APUA-BT)	3	138	772	74.7	1.17	—	—	[191,192]
11-amino-N-(pyridine-2yl)undecanamide modified bentonite (APUA-BT)	5	139	814	74.2	1.13	—	—	[191,192]
		68	1730	113	—	—	—	[193]
graphene nanosheets (GN)	2	86	980	65.1	3.87	—	—	[193]
Ni–Fe layered double hydroxide (Ni–Fe LDH)	2	80	1070	58.9	3.65	—	—	[193]
		49	1261	114	—	—	—	[194]
octaammonium polyhedral oligomeric silsesquioxane-modified montmorillonite (OAPOSS-MMT)	2	42	1207	103	0.99	—	—	[194]
octaammonium polyhedral oligomeric silsesquioxane-modified montmorillonite (OAPOSS-MMT)	4	48	1095	94	1.36	—	—	[194]
octaammonium polyhedral oligomeric silsesquioxane-modified montmorillonite (OAPOSS-MMT)	6	50	982	88	1.69	—	—	[194]
		31	1933	146	—	—	—	[195]
Sodium magadiite (Na-magadiite)	3	39	1283	116	2.38	—	—	[195]
Sodium magadiite reaction with silane coupling agent (S-Na-magadiite)	3	38	1641	120	1.75	—	—	[195]
protonated magadiite reaction with silane coupling agent (S-H-magadiite)	3	38	1416	114	2.14	—	—	[195]
organo-modified magadiite (OM-magadiite)	3	29	1332	105	1.88	—	—	[195]
silane grafting organo modified magadiite (S-OM-magadiite)	3	34	1273	103	2.36	—	—	[195]
	—	32	2572	184	—	—	—	[154]
tetrabromobisphenol-A (TBBA)	17	17	1390	92	1.96	—	—	[154]
		90	1653	130	—	—	—	[196]
graphene sheet (GN)	2	84	1156	108	1.60	—	—	[196]
Ce-doped MnO_2_ (Ce–MnO_2_)	2	79	920	96.7	2.11	—	—	[196]
Ce-doped MnO_2_ decorated graphene sheets (Ce–MnO_2_–GN)	2	100	765	83.8	3.72	—	—	[196]
		89	1473	87.8	—	—	—	[197]
mesoporous silica (m-SiO_2_)	2	107	1191	96.5	1.35	—	—	[197]
Co−Al layered double hydroxide (Co−Al LDH)	2	103	1188	84.3	1.49	—	—	[197]
mesoporous silica@Co−Al layered double hydroxide (m-SiO_2_@Co−Al LDH)	2	110	894	56	3.19	—	—	[197]
		65	1653	130	—	—	—	[198]
Zinc sulfide (ZnS)	2	88	1213	119	2.00	—	—	[198]
graphene sheet (GN)	2	70	1141	108	1.88	—	—	[198]
Zinc sulfide decorated Graphene sheets (ZnS-GN)	2	87	879	94.2	3.47	—	—	[198]
		55	1298	97.6	—	—	—	[199]
hydrated pre-treated sepiolite (sep idra)	2	55	1370	101	0.91	—	—	[199]
hydrated pre-treated sepiolite (sep idra)	5	65	1157	99.5	1.30	—	—	[199]
hydrated pre-treated sepiolite (sep idra)	10	65	1072	95.7	1.45	—	—	[199]
dehydrated pre-treated sepiolite (sep anidra)	2	55	1129	97	1.157	—	—	[199]
dehydrated pre-treated sepiolite (sep anidra)	5	65	1114	107	1.26	—	—	[199]
dehydrated pre-treated sepiolite (sep anidra)	10	65	958	108	1.45	—	—	[199]
		94	1097	119	—	—	—	[158]
expandable graphite (EG)	5	111	463	142	2.34	—	—	[158]
		54	1327	99.1	—	—	—	[159]
chitosan modified montmorillonite intercalation iron compounds (CTS-Fe-OMMT)	3	55	1168	91.4	1.25	—	—	[159]
cetyltrimethylammoniumbromide modified montmorillonite intercalation iron compounds (CTAB-Fe-OMMT)	3	47	975	89.2	1.31	—	—	[159]
		80.4	1111	140	—	—	—	[200]
aminated multiwalled carbon nanotubes supplied by the Polish company (A-MWCNT(Polish))	0.05	72.8	1161	93.6	1.29	—	—	[200]
aminated multiwalled carbon nanotubes supplied by the Polish company (A-MWCNT(Polish))	0.1	68.8	992	93.6	1.43	—	—	[200]
aminated multiwalled carbon nanotubes supplied by the Polish company (A-MWCNT(Polish))	0.5	74	926	96.9	1.59	—	—	[200]
aminated multiwalled carbon nanotubes supplied by the Polish company (A-MWCNT(Polish))	1	71.9	875	92.6	1.72	—	—	[200]
aminated multiwalled carbon nanotubes supplied by the Polish company (A-MWCNT(Polish))	5	78.3	1141	98.9	1.34	—	—	[200]
carboxylated multiwalled carbon nanotubes supplied by the Polish company (C-MWCNT(Polish))	0.05	78.7	1080	101	1.40	—	—	[200]
carboxylated multiwalled carbon nanotubes supplied by the Polish company (C-MWCNT(Polish))	0.1	72.6	1250	100	1.12	—	—	[200]
carboxylated multiwalled carbon nanotubes supplied by the Polish company (C-MWCNT(Polish))	0.5	80.2	1163	98.8	1.35	—	—	[200]
carboxylated multiwalled carbon nanotubes supplied by the Polish company (C-MWCNT(Polish))	1	81.2	945	102	1.63	—	—	[200]
carboxylated multiwalled carbon nanotubes supplied by the Belgian company (C-MWCNT(Belgian))	0.05	76.2	919	96.3	1.66	—	—	[200]
carboxylated multiwalled carbon nanotubes supplied by the Belgian company (C-MWCNT(Belgian))	0.5	67.4	1110	99	1.19	—	—	[200]
carboxyammonium multiwalled carbon nanotubes supplied by the Polish company (CA-MWCNT(Polish))	0.05	83.9	1240	104	1.26	—	—	[200]
carboxyammonium multiwalled carbon nanotubes supplied by the Polish company (CA-MWCNT(Polish))	0.1	73.8	1162	99	1.24	—	—	[200]
carboxyammonium multiwalled carbon nanotubes supplied by the Polish company (CA-MWCNT(Polish))	0.5	76	1095	99.5	1.35	—	—	[200]
carboxyammonium multiwalled carbon nanotubes supplied by the Polish company (CA-MWCNT(Polish))	1	67.3	1192	97.8	1.12	—	—	[200]
carboxyammonium multiwalled carbon nanotubes supplied by the Polish company (CA-MWCNT(Polish))	5	69.7	1198	100	1.12	—	—	[200]
aminated multiwalled carbon nanotubes supplied by the Belgian company (A-MWCNT(Belgian))	0.05	77.4	1314	98.3	1.16	—	—	[200]
aminated multiwalled carbon nanotubes supplied by the Belgian company (A-MWCNT(Belgian))	0.1	80.2	1225	98.6	1.28	—	—	[200]
aminated multiwalled carbon nanotubes supplied by the Belgian company (A-MWCNT(Belgian))	0.5	56.6	1005	62.4	1.75	—	—	[200]
		66	934	95	—	—	—	[201]
graphene oxide (GNO)	1	76	811	133	0.94	—	—	[201]
		41	1222	159	—	—	—	[160]
onium ion modified nanoclay (I.30E)	3	32	1274	154	0.77	—	—	[160]
	0	101	1348	87.1	—	—	—	[202]
molybdenum disulfide (MoS_2_)	2	96	1076	75.7	1.37	—	—	[202]
graphene (GN)	2	92	965	70.1	1.58	—	—	[202]
molybdenum disulfide modified graphene (MoS_2_-GN)	2	90	730	65.1	2.20	—	—	[202]
	0	47	1630	82.3	—	—	—	[161]
graphene oxide(GNO)	1	41	1426	76.8	1.06	—	—	[161]
epoxy resin modified with (3-isocyanatopropyl)-triethoxysilane	0	93	1331	63.8	—	—	—	[203]
hydroxylated hexagonal boron nitride (BNO)	1	113	860	56.3	2.13	—	—	[203]
hydroxylated hexagonal boron nitride (BNO)	3	117	765	55.5	2.51	—	—	[203]
	—	42	385	21.8	—	27.5	—	[163,164]
cellulosic fibre containing polysilicic acid (Vis) ^a^	4.7	41	329	19.4	1.28	28.1	—	[163,164]
phenol–formaldehyde fibers (Ky) ^a^	4.7	51	367	28.8	0.96	27.7	—	[163,164]
		44	818	28.8	—	—	—	[204]
Nanoclay (clay) ^b^	1	32	558	26.4	1.16	—	—	[204]
Nanoclay (clay) ^b^	3	32	570	25.5	1.18	—	—	[204]
Nanoclay (clay) ^b^	5	32	533	24.8	1.29	—	—	[204]
		28	349	20.4	—	—	—	[150]
layered double hydroxide (LDH) ^c^	5	22	343	21.9	0.74	—	—	[150]
Hydrogenated fatty acid modified layered double hydroxide (OLDH) ^c^	5	21	310	23	0.74	—	—	[150]
carbon nanotube (CNT) ^c^	1	27	396	22.7	0.76	—	—	[150]
chemical treatment carbon nanotube (CCNT) ^c^	1	26	411	21.7	0.74	—	—	[150]
thermal treatment carbon nanotube (TCNT) ^c^	1	27	471	22.2	0.65	—	—	[150]
aluminium trihydroxide (ATH) ^c^	5	22	417	22.6	0.59	—	—	[150]
	0	33	520	29.4	—	—	—	[205]
magnesium hydroxide (Mg(OH)_2_) ^d^	1	28	518	37.4	0.67	—	—	[205]
magnesium hydroxide (Mg(OH)_2_) ^d^	7.5	30	550	28.4	0.89	—	—	[205]
magnesium hydroxide (Mg(OH)_2_) ^d^	15	30	392	31.2	1.13	—	—	[205]
magnesium hydroxide (Mg(OH)_2_) ^d^	25	35	476	41.7	0.81	—	—	[205]
aluminum hydroxide (Al(OH)_3_) ^d^	1	28	456	37.3	0.76	—	—	[205]
aluminum hydroxide (Al(OH)_3_) ^d^	7.5	28	585	35.3	0.62	—	—	[205]
aluminum hydroxide (Al(OH)_3_) ^d^	15	26	451	32.8	0.81	—	—	[205]
aluminum hydroxide (Al(OH)_3_) ^d^	25	32	396	31.6	1.18	—	—	[205]
Zinc borate (ZB) ^d^	1	26	572	35.9	0.58	—	—	[205]
Zinc borate (ZB) ^d^	7.5	32	427	42.7	0.81	—	—	[205]
Zinc borate (ZB) ^d^	15	27	458	36.3	0.75	—	—	[205]
Zinc borate (ZB) ^d^	25	37	352	30.6	1.59	—	—	[205]
		46	568	23.2	—	—	—	[206]
Single-walled carbon nanotube Buckypaper (SWCNT-BP) ^e^	1.06	50	526	24.5	1.11	—	—	[206]
multiwalled carbon nanotube Buckypaper (MWCNT-BP) ^e^	1.34	64	258	13.2	5.38	—	—	[206]
carbon nanofiber (CNF) ^e^	1.57	59	508	24.8	1.34	—	—	[206]
	0	39	456	38	—	—	—	[167]
cellulosic fibre containing polysilicic acid (Vis) ^f^	5	46	451	37.2	1.21	—	—	[167]
cellulosic fibre containing polysilicic acid (Vis) ^f^	10	58	434	36.3	1.63	—	—	[167]
cellulosic fibre containing polysilicic acid (Vis) ^f^	15	55	321	31.1	2.44	—	—	[167]
		46	568	23.2	—	—	—	[207]
Single-walled carbon nanotube Buckypaper (SWCNT-BP) ^g^	1.06	50	526	24.5	1.11	—	—	[207]
multiwalled carbon nanotube Buckypaper (MWCNT-BP) ^g^	1.34	64	258	13.2	5.38	—	—	[207]
		125	857	50	—	—	—	[174]
Trisilanolisobutyl Polyhedral oligomeric silsesquioxane (T8POSS) ^h^	5	121	420	32	3.08	—	—	[174]
triglycidyl isocyanurate (TGIC) ^h^	5	108	620	47	1.27	—	—	[174]

^a^ Matrix: eight layers of woven E-glass reinforced film of multifunctional epoxy resin; ^b^ Matrix: six layers of biaxial E-glass fabric reinforced epoxy; ^c^ Matrix: carbon fiber reinforced epoxy resin; ^d^ Matrix: eight plies of carbon fiber reinforced system HexFlow RTM6 (matrix) and HexForce G0939 (fabric); ^e^ Matrix: six layers of IM-7 carbon fiber fabrics reinforced epoxy; ^f^ Matrix: eight layers of woven E-glass reinforced epoxy; ^g^ Matrix: six layers of IM-7 carbon fiber fabrics reinforced epoxy; ^h^ Matrix: eight layers of woven glass Fiber Reinforced epoxy.

**Table 3:** The flame retardancy performance of epoxy containing combinatory flame retardants in terms of FRI (* the name and percentage of incorporated flame retardant is given after each epoxy resin). Notes *a* to *i* on the bottom of the table are representative of composite systems containing woven or nonwoven fibers.

Epoxy Resins and Incorporated P/NP FR *	wt.%	TTI (s)	pHRR (kW·m^−^^2^)	THR (MJ·m^−^^2^)	FRI	LOI	UL94	Ref.
	0	32	827	116	—	21.8	NR	[28]
phenethyl-bridged 9,10-dihydro-9-oxa-10-phosphaphenanthrene-10-oxide derivative/graphene nanosheet (DiDOPO/GN)	3	51	374	99	4.13	32.2	V-0	[28]
	0	32	781	107	—	21.8	NR	[29]
phenethyl-bridged 9,10-dihydro-9-oxa-10-phosphaphenanthrene-10-oxide derivative/multiwalled carbon nanotube (DiDOPO/MWCNT)	10.8	47	352	72	4.84	38.6	V-0	[29]
	0	32	781	107	—	21.8	NR	[30]
phenethyl-bridged 9,10-dihydro-9-oxa-10-phosphaphenanthrene-10-oxide derivative/Organically modified montmorillonite (DiDOPO/OMMT)	7	46	396	95	3.19	32.2	V-0	[30]
	0	32	781	107	—	21.8	NR	[31]
phenethyl-bridged 9,10-dihydro-9-oxa-10-phosphaphenanthrene-10-oxide derivative/organomodified magnesium aluminium layered double hydroxide (DiDOPO/OLDH)	1	41	437	142	1.73	25.2	V-0	[31]
phenethyl-bridged 9,10-dihydro-9-oxa-10-phosphaphenanthrene-10-oxide derivative/organomodified magnesium aluminium layered double hydroxide (DiDOPO/OLDH)	5	44	420	120	2.28	27.8	V-0	[31]
phenethyl-bridged 9,10-dihydro-9-oxa-10-phosphaphenanthrene-10-oxide derivative/organomodified magnesium aluminium layered double hydroxide (DiDOPO/OLDH)	10	46	406	82	3.61	31.5	V-0	[31]
	0	30	1293	86.9	—	19.2	HB	[208]
IFR: Ammonium polyphosphate & pentaerythritol & melamine(APP & PER & MEL/5:3:2) (IFR)	40	10	314	51	2.34	29.1	V-0	[208]
IFR: Ammonium polyphosphate & pentaerythritol & melamine(APP & PER & MEL/5:3:2)/Chicken eggshell (IFR/CES)	40	22	266	45.9	6.75	29.6	V-0	[208]
IFR: Ammonium polyphosphate & pentaerythritol & melamine(APP & PER & MEL/5:3:2)/Chicken eggshell (IFR/CES)	40	12	235	41.3	4.63	30.4	V-0	[208]
IFR: Ammonium polyphosphate & pentaerythritol & melamine(APP & PER & MEL/5:3:2)/Chicken eggshell (IFR/CES)	40	23	181	33	14.4	31.5	V-0	[208]
IFR: Ammonium polyphosphate & pentaerythritol & melamine(APP & PER & MEL/5:3:2)/Chicken eggshell (IFR/CES)	40	20	201	38	9.81	30.7	V-0	[208]
Waterborne EP resin	0	25	344	18.3	—	19.3	NR	[41]
9,10-dihydro-9-oxa-10-phosphaphenanthrene-10-oxide/phosphated K-carrageenan (DOPO/P-KC)	30	13	176	13.3	1.39	27.1	V-0	[41]
9,10-dihydro-9-oxa-10-phosphaphenanthrene-10-oxide/phosphated K-carrageenan (DOPO/P-KC)	30	15	131	12.3	2.34	28.2	V-0	[41]
9,10-dihydro-9-oxa-10-phosphaphenanthrene-10-oxide/phosphated K-carrageenan (DOPO/P-KC)	30	20	197	14.5	1.76	25	V-1	[41]
	0	49	1247	49.8	—	22.5	NR	[209]
microencapsulated ammonium polyphosphate/pentaerythritol (mAPP/PER)	10	27	961	39.9	0.89	29.9	NR	[209]
microencapsulated ammonium polyphosphate/regenerated cotton cellulose (mAPP/RCC)	10	30	1055	40.5	0.89	24.1	NR	[209]
microencapsulated ammonium polyphosphate/oxidized regenerated cotton cellulose (mAPP/ORCC)	10	29	554	20.9	3.17	29.5	V-0	[209]
	0	21	490	103	—	18.3	NR	[210]
2,6,7-trioxa-1-phosphabicyclo-[2.2.2]-octane-4-methanol-trimellitic anhydride/melamine cyanurate (PEPA–TMA/MCA)	18	17	378	90.4	1.20	28.9	V-1	[210]
2,6,7-trioxa-1-phosphabicyclo-[2.2.2]-octane-4-methanol-trimellitic anhydride/melamine cyanurate (PEPA–TMA/MCA)	24	15	221	57.6	2.84	29.8	V-0	[210]
2,6,7-trioxa-1-phosphabicyclo-[2.2.2]-octane-4-methanol-trimellitic anhydride/melamine cyanurate (PEPA–TMA/MCA)	30	12	296	74.8	1.31	29.1	V-1	[210]
	0	71	1146	56	—	21.2	NR	[170]
zeolitic imidazolate framework8/MgAl-layered double hydroxide (ZIF8/MgAl-LDH)	2	64	742	42	1.86	24	NR	[170]
zeolitic imidazolate framework67/MgAl-layered double hydroxide (ZIF67/MgAl-LDH)	2	65	719	41	1.99	24.2	NR	[170]
	0	61	1208	77.3	—	22.5	NR	[52]
triazine-based flame retardant/9,10-Dihydro-9-oxa-10-phosphaphenanthrene-10-oxide (TAT/DOPO)	20	44	849	74.3	1.07	29.5	NR	[52]
triazine-based flame retardant/9,10-Dihydro-9-oxa-10-phosphaphenanthrene-10-oxide (TAT/DOPO)	20	44	682	64.5	1.53	34	V-1	[52]
triazine-based flame retardant/9,10-Dihydro-9-oxa-10-phosphaphenanthrene-10-oxide (TAT/DOPO)	20	47	558	56.3	2.29	36	V-0	[52]
triazine-based flame retardant/9,10-Dihydro-9-oxa-10-phosphaphenanthrene-10-oxide (TAT/DOPO)	20	41	500	48.5	2.59	38.6	V-0	[52]
triazine-based flame retardant/hexa-phenoxy-cyclotriphosphazene (TAT/HPCP)	20	46	774	72.3	1.26	30.1	NR	[52]
triazine-based flame retardant/hexa-phenoxy-cyclotriphosphazene (TAT/HPCP)	20	43	598	59.3	1.86	33.5	V-1	[52]
triazine-based flame retardant/hexa-phenoxy-cyclotriphosphazene (TAT/HPCP)	20	48	484	52.6	2.89	37.3	V-0	[52]
triazine-based flame retardant/hexa-phenoxy-cyclotriphosphazene (TAT/HPCP)	20	48	437	47.8	3.52	39.6	V-0	[52]
		53	1121	102	—	20	NR	[55]
ethanediamine-modified ammonium polyphosphate/Cuprous oxide (EDA-APP/Cu_2_O)	21	62	364	64	5.74	33.5	V-0	[55]
		45	1091	83	—	22.8	NR	[56]
hexakis(4-boronic acid-phenoxy)-cyclophosphazene/magnesium hydroxide (CP-6B/MH)	3.5	49	535	67	2.75	31.9	V-0	[56]
		93.6	851	91.7	—	19.7	NR	[211]
IFR:ammonium polyphosphate & pentaerythritol(APP & PER/3:1) (IFR)	20	42.8	266	89.7	1.50	27.3	V-1	[211]
IFR:ammonium polyphosphate & pentaerythritol(APP & PER/3:1)/Hollow glass microsphere (IFR/HGM)	20	55.4	246	59.7	3.15	28.8	V-1	[211]
IFR:ammonium polyphosphate & pentaerythritol(APP & PER/3:1)/Hollow glass microsphere (IFR/HGM)	20	50.6	210	59.6	3.36	29.1	V-1	[211]
IFR:ammonium polyphosphate & pentaerythritol(APP & PER/3:1)/Hollow glass microsphere (IFR/HGM)	20	74.9	178	44.8	7.85	34.7	V-0	[211]
IFR:ammonium polyphosphate & pentaerythritol(APP & PER/3:1)/Hollow glass microsphere (IFR/HGM)	20	51.2	215	54.3	3.67	31.4	V-0	[211]
		43	469	66.2	—	24.7	NR	[58]
Ammonium polyphosphate/poly(4,40-diamino diphenyl sulfone 2,6,7-trioxa-1-phosphabicyclo[2.2.2]octane-4-methanol-substituted phosphoramide) (APP/PSA)	10	34	132	21.3	8.73	32	V-0	[58]
		29	1340	36.3	—	22.5	NR	[212]
microencapsulated ammonium polyphosphate/pentaerythritol (MFAPP/PER)	12.5	24	422	20.6	4.63	24.9	NR	[212]
microencapsulated ammonium polyphosphate/corn starch (MFAPP/ST)	12.5	24	457	15.2	5.80	30.1	V-0	[212]
microencapsulated ammonium polyphosphate/oxidized corn starch (MFAPP/OST)	12.5	22	400	13.4	6.88	29.5	V-0	[212]
		58	1208	80.6	—	22.5	NR	[66]
expandable graphite/9,10-dihydro-9-oxa-10-phosphaphenanthrene-10-oxide (EG/DOPO)	20	48	236	48.4	7.05	35	V-1	[66]
expandable graphite/9,10-dihydro-9-oxa-10-phosphaphenanthrene-10-oxide (EG/DOPO)	20	48	296	48.8	5.58	38	V-0	[66]
expandable graphite/9,10-dihydro-9-oxa-10-phosphaphenanthrene-10-oxide (EG/DOPO)	20	48	405	50	3.98	42	V-0	[66]
expandable graphite/9,10-dihydro-9-oxa-10-phosphaphenanthrene-10-oxide (EG/DOPO)	20	48	442	51.4	3.55	41.5	V-0	[66]
expandable graphite/hexa-phenoxy-cyclotriphosphazene (EG/HPCP)	20	48	259	49.7	6.26	33.5	V-1	[66]
expandable graphite/hexa-phenoxy-cyclotriphosphazene (EG/HPCP)	20	48	340	48	4.94	36	V-0	[66]
expandable graphite/hexa-phenoxy-cyclotriphosphazene (EG/HPCP)	20	48	809	50.6	1.97	40.5	V-0	[66]
expandable graphite/hexa-phenoxy-cyclotriphosphazene (EG/HPCP)	20	48	760	42.2	2.51	39	V-0	[66]
		57	1557	94.5	—	24.5	NR	[67]
nucleophilic substitution reaction between N-(4-hydroxyphenyl) maleimide & cyanuric chloride/9,10-dihydro-9-oxa-10-phosphaphenanthrene-10-oxide (TMT/DOPO)	11	45	1210	74.7	1.29	34	V-1	[67]
nucleophilic substitution reaction between N-(4-hydroxyphenyl) maleimide & cyanuric chloride/9,10-dihydro-9-oxa-10-phosphaphenanthrene-10-oxide (TMT/DOPO)	12.3	46	1085	70.3	1.56	36.5	V-0	[67]
nucleophilic substitution reaction between N-(4-hydroxyphenyl) maleimide & cyanuric chloride/9,10-dihydro-9-oxa-10-phosphaphenanthrene-10-oxide (TMT/DOPO)	13.7	47	1105	70.8	1.55	38	V-0	[67]
nucleophilic substitution reaction between N-(4-hydroxyphenyl) maleimide & cyanuric chloride/9,10-dihydro-9-oxa-10-phosphaphenanthrene-10-oxide (TMT/DOPO)	15	44	980	61	1.90	40.3	V-0	[67]
		56	1420	116	—	26.2	NR	[75]
9,10-dihydro-9-oxa-10-phosphaphenanthrene 10-oxide/aluminum poly-hexamethylenephosphinate (DOPO/APHP)	6	50	539	63	4.33	39.3	V-1	[75]
9,10-dihydro-9-oxa-10-phosphaphenanthrene 10-oxide/aluminum poly-hexamethylenephosphinate (DOPO/APHP)	6	46	510	58	4.57	39.5	V-0	[75]
		56	1420	140	—	26	NR	[77]
reaction between triallyl isocyanurate & 9,10-dihydro-9-oxa-10-phosphaphenanthrene-10-oxide/organically modified montmorillonite (TAD/OMMT)	5	41	961	108	1.40	36.9	V-0	[77]
		82	685	145	—	21.3	NR	[213]
flame retardant containing phosphorus & 4-tert-butylcalix[4]arene/ammonium polyphosphate (FR/APP)	30	92	332	108	3.11	27.4	V-1	[213]
flame retardant containing phosphorus & 4-tert-butylcalix[4]arene/ammonium polyphosphate (FR/APP)	30	91	361	82	3.73	28.6	V-1	[213]
flame retardant containing phosphorus & 4-tert-butylcalix[4]arene/ammonium polyphosphate (FR/APP)	30	115	229	74	8.22	29.3	V-0	[213]
flame retardant containing phosphorus & 4-tert-butylcalix[4]arene/ammonium polyphosphate (FR/APP)	30	100	203	74	8.07	30.8	V-0	[213]
		62	840	84	—	23	V-1	[89]
amine-terminated cyclophosphazene/3-aminopropyltrimethoxy silane-functionalized rice husk ash (ATCP/FRHA)	16	56	542	56	2.10	44	V-0	[89]
amine-terminated cyclophosphazene/3-aminopropyltrimethoxy silane-functionalized rice husk ash (ATCP/FRHA)	18	69	427	42	4.38	51	V-0	[89]
amine-terminated cyclophosphazene/3-aminopropyltrimethoxy silane-functionalized rice husk ash (ATCP/FRHA)	20	77	340	30	8.59	62	V-0	[89]
		57	713	64	—	—	—	[90]
amine-terminated cyclophosphazene/3-aminopropyltrimethoxy silane-functionalized rice husk ash (ATCP/FRHA)	16	48	435	51	1.73	39	V-0	[90]
amine-terminated cyclophosphazene/3-aminopropyltrimethoxy silane-functionalized rice husk ash (ATCP/FRHA)	18	45	374	43	2.24	45	V-0	[90]
amine-terminated cyclophosphazene/3-aminopropyltrimethoxy silane-functionalized rice husk ash (ATCP/FRHA)	20	40	289	31	3.57	51	V-0	[90]
		50	860	112	—	23	NR	[93]
Ammonium polyphosphate/montmorillonite (APP/MMT)	10	53	524	50	3.90	28	V-0	[93]
		50	860	133	—	23	NR	[94]
9,10-dihydro-9-oxa-10-phosphaphenanthrene-10-oxide/Montmorillonite (DOPO/MMT)	6	52	473	76	3.31	33	V-1	[94]
		65	966	96	—	22.5	NR	[95]
bisphenol-A bis(diphenyl phosphate)/aluminum poly-hexamethylenephosphinate (BDP/PHP)	10	51	672	86	1.26	35	V-0	[95]
		45	855	112	—	3	3.2	[102]
octaphenyl polyhedral oligomeric silsesquioxane/9,10-dihydro-9-oxa-10-phosphaphenanthrene-10-oxide (OPS/DOPO)	5	54	603	89	2.14	29	V-1	[102]
		45	855	112	—	25	NR	[103]
9,10-dihydro-9-oxa-10-phosphaphenanthrene-10-oxide/Octaphenyl silsesquioxane (DOPO/OPS)	5.2	51	557	95	2.05	31.1	V-0	[103]
9,10-dihydro-9-oxa-10-phosphaphenanthrene-10-oxide/Polyphenyl silsesquioxane (DOPO/PPSQ)	5.2	49	895	100	1.17	31.2	NR	[103]
		45	855	112	—	25	NR	[104]
9,10-dihydro-9-oxa-10-phosphaphenanthrene-10-oxide/Octaphenyl silsesquioxane (DOPO/OPS)	5.2	51	557	95	2.05	31.1	V-0	[104]
9,10-dihydro-9-oxa-10-phosphaphenanthrene-10-oxide/Octaaminophenylsilsesquioxane (DOPO/OAPS)	5.4	53	645	102	1.71	33.8	V-1	[104]
		50	860	112	—	3	3.2	[105]
octaphenyl polyhedral oligomeric silsesquioxane/ 9,10-dihydro-9-oxa-10-phosphaphenanthrene-10-oxide (OPS/DOPO)	5	58	540	82	2.52	31	V-0	[105]
		50	860	112	—	—	—	[106]
Octaphenyl polyhedral oligomeric silsesquioxane/1-oxo-4-hydroxymethyl-2,6,7-trioxa-l-phosphabicyclo[2.2.2] octane (OPS/PEPA)	4.7	52	524	84	2.28	25.5	NR	[106]
Octaphenyl polyhedral oligomeric silsesquioxane/Ammonium polyphosphate (OPS/APP)	3.5	63	584	101	2.06	24.6	NR	[106]
Octaphenyl polyhedral oligomeric silsesquioxane/9,10-dihydro-9-oxa-10-phosphaphenanthrene-10-oxide (OPS/DOPO)	5.2	55	548	83	2.33	30.8	V-1	[106]
		64	821	94	—	23.2	NR	[115]
polyhedral oligomeric octadiphenylsulfonylsilsesquioxane/9, 10-Dihydro-9-oxa-10-phosphaphenanthrene-10-oxide (ODPSS/DOPO)	5	57	438	69	2.27	29.8	V-0	[115]
		20	662	88.6	—	20.5	NR	[214]
bis(diphenyl phosphate) oligomer/polyphosphoric acid (BBO/PPA)	20	30	224	63.2	6.21	26	V-0	[214]
		108	1634	78	—	19.8	NR	[174]
Trisilanolisobutyl Polyhedral oligomeric silsesquioxane/triglycidyl isocyanurate (T8POSS/TGIC)	10	88	944	58	1.90	20.9	NR	[174]
		64	939	179	—	19.6	NR	[117]
ammonium polyphosphate/metal compounds (APP/CoSA)	5	65	310	95	5.80	29.4	V-0	[117]
		53	1262	84.7	—	25	NR	[118]
cardanol derived benzoxazine monomer/boron-doped graphene (CBz/BGN)	10	49	870	75.9	1.50	30	V-0	[118]
cardanol derived benzoxazine monomer/boron-doped graphene (CBz/BGN)	15	52	650	74.4	2.17	33	V-0	[118]
cardanol derived benzoxazine monomer/boron-doped graphene (CBz/BGN)	20	56	716	78.7	2.00	33	V-0	[118]
		21	1910	84.4	—	22.1	NR	[120]
melamine coated ammonium polyphosphate/layered double hydroxide (Mel-APP/LDH)	20	20	240	30.3	21.10	33.2	V-0	[120]
melamine coated ammonium polyphosphate/halloysite nano-tube (Mel-APP/HNT)	20	20	246	26.2	23.90	32.7	V-0	[120]
epoxy novolac resin	0	51	682	110	—	—	NR	[124]
oligo[DOPAc-2-tris(acryloyloxy)ethyl isocyanurate] /melamine polyphosphate (oDOPI/MPP)	32.8	48	341	85	2.44	—	V-0	[124]
boehmite/oligo[DOPAc-2-tris(acryloyloxy)ethyl isocyanurate] (AlO(OH)/oDOPI)	41.1	71	319	74	4.42	—	V-0	[124]
melamine polyphosphate/phosphazene (MPP/PZ)	16.5	50	310	82	2.89	—	V-0	[124]
boehmite/phosphazene (AlO(OH)/PZ)	33.1	66	435	79	2.83	—	V-0	[124]
	0	50	986	91	—	—	NR	[125]
aluminum hypophosphite/activated carbon spheres@SnO_2_@NiO hybrid (AHP/ACS@SnO_2_@NiO)	5	54	714	76	1.78	—	V-0	[125]
		23	1910	61	—	—	NR	[126]
Melamine coated ammonium polyphosphate/Talc (Mel-APP/Talc)	29.7	28	357	24	16.60	—	V-0	[126]
		54	1068	75.8	—	—	HB	[127]
melamine polyphosphate/melamine poly(zinc phosphate) (MPP/MPZnP)	20	38	207	51.1	5.39	—	V-1	[127]
diethyl aluminum phosphinate/melamine poly(zinc phosphate) (AlPi-Et/MPZnP)	20	43	405	51.2	3.11	—	HB	[127]
6H-dibenz[c,e][1,2] oxaphosphorin-6-propanoic acid, butyl ester, 6-oxide/melamine poly(zinc phosphate) (DOPAc-Bu/MPZnP)	20	42	329	57.6	3.32	—	V-1	[127]
boehmite/melamine poly(zinc phosphate) (AlO(OH)/MPZnP)	20	43	438	57.2	2.57	—	HB	[127]
amorphous silicon dioxide/melamine poly(zinc phosphate) (MPZnP/SiO_2_)	20	37	525	62.4	1.69	—	HB	[127]
melamine polyphosphate/melamine poly(zinc phosphate) (MPP/MPZnP)	20	41	211	32.5	8.96	—	V-0	[127]
diethyl aluminum phosphinate/melamine poly(zinc phosphate) (AlPi-Et/MPZnP)	20	41	435	53.8	2.63	—	V-1	[127]
6H-dibenz[c,e][1,2] oxaphosphorin-6-propanoic acid, butyl ester, 6-oxide/melamine poly(zinc phosphate) (DOPAc-Bu/MPZnP)	20	41	412	52.1	2.86	—	HB	[127]
boehmite/melamine poly(zinc phosphate) (AlO(OH)/MPZnP)	20	43	575	57.9	1.94	—	HB	[127]
amorphous silicon dioxide/melamine poly(zinc phosphate) (SiO_2_/MPZnP)	20	37	681	65.6	1.24	—	HB	[127]
		63	1321	157	—	—	NR	[129]
hexaphenoxycyclotriphosphazene/octapropylglycidylether polyhedral oligomeric silsesquioxane (HPCTP/OGPOSS)	15	58	707	123	2.20	—	V-0	[129]
hexaphenoxycyclotriphosphazene/octapropylglycidylether polyhedral oligomeric silsesquioxane (HPCTP/OGPOSS)	15	56	581	110	2.88	—	V-0	[129]
hexaphenoxycyclotriphosphazene/octapropylglycidylether polyhedral oligomeric silsesquioxane (HPCTP/OGPOSS)	15	56	560	105	3.14	—	V-0	[129]
		100	733	141	—	21	HB	[130]
Tetraphenylphosphonium modified montmorillonite/Silicate glass (CP/TPP-MMT)	15	101	353	131	2.26	25	HB	[130]
		47	891	151	—	21	HB	[130]
Tetraphenylphosphonium modified montmorillonite/Silicate glass (CP/TPP-MMT)	15	48	474	130	2.23	25	HB	[130]
		22	1196	147	—	21	HB	[130]
Tetraphenylphosphonium modified montmorillonite/Silicate glass (CP/TPP-MMT)	15	22	617	130	2.19	25	HB	[130]
	0	69	1150	54.7	—	22	—	[176]
molybdenum disulfide/titanium dioxide nanotube (MoS_2_/TNT)	2	56	742	38.6	1.78	26	—	[176]
		24	1002	104	—	18	—	[215]
Ammonium polyphosphate/Pentaerythritol modified halloysite tube (APP/PER-HNT)	25	33	562	51.8	4.93	24.8	—	[215]
		54	1068	76	—	21	—	[147]
melamine poly(magnesium phosphate)/aluminium diethylphosphinate (S600/AlPi)	20	44	479	46	3.00	30.4	—	[147]
melamine poly(magnesium phosphate)/boehmite (S600/AlO(OH))	20	38	437	55	2.38	28.9	—	[147]
melamine poly(magnesium phosphate)/melamine polyphosphate (S600/MPP)	20	39	208	54	5.22	28.4	—	[147]
		86	1650	213	—	20.2	—	[136]
3-((Methoxydiphenylsilyl) oxy)-9-methyl-2, 4, 8, 10-tetraoxa-3, 9-diphosphaspiro [5. 5] undecane 3, 9-dioxide/Mono (4, 6-diamino-1, 3, 5-triazin-2-aminium) (2, 4, 8, 10-tetraoxa-3, 9-diphosphaspiro [5. 5] undecane-3, 9-bis (olate) 3, 9-dioxide) (SDPS/SPDM)	10.4	62	1122	207	1.09	30.8	—	[136]
	0	70	1491	81	—	19	NR	[47]
aluminum diethyl phosphinate/Melamine polyphosphate (AlPi/MPP)	7	61	505	48	4.34	—	—	[47]
aluminum diethyl phosphinate/Melamine polyphosphate/aluminum oxide (AlPi/MPP/Al_2_O_3_)	7	66	533	58	3.68	—	—	[47]
	0	25	1113	223	—	—	—	[139]
ammonium polyphosphate/char sulfonic acid (APP/CSA)	10	24	672	127	2.78	—	—	[139]
ammonium polyphosphate/char sulfonic acid (APP/CSA)	10	23	665	107	3.21	—	—	[139]
ammonium polyphosphate/char sulfonic acid (APP/CSA)	10	27	698	137	2.81	—	—	[139]
	0	117	1184	95.3	—	—	—	[182]
Boron Nitride with D50 = 12 μm/Boron Nitride with D50 = 2 μm (BN 12 μm/BN 2 μm)	45	164	918	75.7	2.28	—	—	[182]
Boron Nitride with D50 = 12 μm/Boehmite with D50 = 2 μm (BN 12 μm/BT 2 μm)	45	163	729	65.1	3.31	—	—	[182]
		60	923	124	—	—	—	[216]
IFR: ammonium polyphosphate & pentaerythritol(APP & PER/3:1) (IFR)	30	64	285	64.1	6.69	—	—	[216]
IFR: ammonium polyphosphate & pentaerythritol(APP & PER/3:1)/ferric phosphate (IFR/FeP)	30	46	170	56	9.23	—	—	[216]
IFR:ammonium polyphosphate & pentaerythritol(APP & PER/3:1)/ferric phosphate (IFR/FeP)	30	42	185	49.3	8.80	—	—	[216]
IFR: ammonium polyphosphate & pentaerythritol(APP & PER/3:1)/ferric phosphate (IFR/FeP)	30	39	167	39.7	11.20	—	—	[216]
IFR: ammonium polyphosphate & pentaerythritol(APP & PER/3:1)/ferric phosphate (IFR/FeP)	30	41	180	44.6	9.76	—	—	[216]
		62	913	155	—	—	—	[217]
IFR: ammonium polyphosphate & pentaerythritol(APP & PER/3:1) (IFR)	30	49	260	56	7.68	—	—	[217]
IFR: ammonium polyphosphate & pentaerythritol(APP & PER/3:1)/ferrite yellow: goethite (IFR/αFeOOH)	30	46	172	47	13.00	—	—	[217]
IFR: ammonium polyphosphate & pentaerythritol(APP & PER/3:1)/ferrite yellow: goethite (IFR/αFeOOH)	30	53	166	36	20.20	—	—	[217]
IFR: ammonium polyphosphate & pentaerythritol(APP & PER/3:1)/ferrite yellow: goethite (IFR/αFeOOH)	30	50	196	40	14.60	—	—	[217]
IFR: ammonium polyphosphate & pentaerythritol(APP & PER/3:1)/ferrite yellow: goethite (IFR/αFeOOH)	30	52	217	74	7.39	—	—	[217]
		60	923	124	—	—	—	[218]
IFR: ammonium polyphosphate & pentaerythritol(APP & PER/3:1) (IFR)	30	49	285	64.1	5.12	—	—	[218]
IFR: ammonium polyphosphate & pentaerythritol(APP & PER/3:1)/iron oxide brown (IFR/iron oxide brown)	30	34	167	38.3	10.20	—	—	[218]
IFR: ammonium polyphosphate & pentaerythritol(APP & PER/3:1)/iron oxide brown (IFR/iron oxide brown)	30	45	126	31	22.00	—	—	[218]
IFR: ammonium polyphosphate & pentaerythritol(APP & PER/3:1)/iron oxide brown (IFR/iron oxide brown)	30	48	124	29.3	25.20	—	—	[218]
IFR: ammonium polyphosphate & pentaerythritol(APP & PER/3:1)/iron oxide brown (IFR/iron oxide brown)	30	53	163	43.2	14.40	—	—	[218]
		68	1730	113	—	—	—	[193]
Ni–Fe layered double hydroxide/graphene nanosheets (Ni–Fe LDH/GN)	2	89	678	44.2	8.55	—	—	[193]
Epoxy acrylic		32	223	30.8	—	—	—	[152]
ammonium polyphosphate/pentaerythritol (APP/PER)	30	61	188	25.2	2.77	—	—	[152]
		70	934	124	—	—	—	[219]
IFR: ammonium polyphosphate & pentaerythrite(APP & PER/3:1) (IFR)	30	70	282	64	6.42	—	—	[219]
IFR: ammonium polyphosphate & pentaerythrite(APP & PER/3:1)/organic-modified iron–montmorillonite (IFR/Fe-OMMT)	30	20	243	70	1.95	—	—	[219]
IFR: ammonium polyphosphate & pentaerythrite(APP & PER/3:1)/organic-modified iron–montmorillonite (IFR/Fe-OMMT)	30	15	153	54	3.00	—	—	[219]
IFR: ammonium polyphosphate & pentaerythrite(APP & PER/3:1)/organic-modified iron–montmorillonite (IFR/Fe-OMMT)	30	30	154	68	4.74	—	—	[219]
IFR: ammonium polyphosphate & pentaerythrite(APP & PER/3:1)/organic-modified iron–montmorillonite (IFR/Fe-OMMT)	30	15	194	65	1.97	—	—	[219]
		41	1222	159	—	—	—	[160]
ammonium polyphosphate/onium ion modified nanoclay (APP/I.30E)	23	149	363	92	21.10	—	—	[160]
	0	21	454	36.2	—	22.1	NR	[120]
melamine coated ammonium polyphosphate/layered double hydroxide (Mel-APP/LDH) ^a^	9.55	21	259	22.6	2.81	31.7	V-1	[120]
melamine coated ammonium polyphosphate/halloysite nano-tube (Mel-APP/HNT) ^a^	9.61	22	262	18.4	3.57	31.4	V-1	[120]
		24	451	37	—	—	NR	[126]
Melamine coated ammonium polyphosphate/Talc (Mel-APP/Talc) ^b^	14.8	21	169	16	5.40	—	NR	[126]
	—	42	385	21.8	—	27.5	—	[163,164]
IFR contains melamine phosphate/cellulosic fibre containing polysilicic acid (IFR/Vis) ^c^	10	38	262	17.9	1.62	36.2	—	[163,164]
IFR contains melamine phosphate/phenol–formaldehyde fibers (Ky/IFR) ^c^	10	55	354	23.2	1.34	30.2	—	[163,164]
	0	33	520	29.4	—	—	—	[205]
Zinc borate/magnesium hydroxide (ZB/Mg(OH)_2_) ^d^	1	32	552	41.8	0.64	—	—	[205]
Zinc borate/magnesium hydroxide (ZB/Mg(OH)_2_) ^d^	7.5	37	483	37.4	0.95	—	—	[205]
Zinc borate/magnesium hydroxide (ZB/Mg(OH)_2_) ^d^	15	38	439	35.4	1.13	—	—	[205]
Zinc borate/magnesium hydroxide (ZB/Mg(OH)_2_) ^d^	25	40	380	27.2	1.79	—	—	[205]
Zinc borate/aluminum hydroxide (ZB/Al(OH)_3_) ^d^	1	33	525	35	0.83	—	—	[205]
Zinc borate/aluminum hydroxide (ZB/Al(OH)_3_) ^d^	7.5	36	480	37.4	0.93	—	—	[205]
Zinc borate/aluminum hydroxide (ZB/Al(OH)_3_) ^d^	15	27	439	37.2	0.77	—	—	[205]
Zinc borate/aluminum hydroxide (ZB/Al(OH)_3_) ^d^	25	30	409	37.7	0.90	—	—	[205]
		44	853	51.9	—	—	—	[166]
melamine phosphate/Graphene (MP/GN) ^e^	5	36	483	47.9	1.57	—	—	[166]
9,10-Dihydro-9-oxa-10-phosphaphenanthrene-10-oxide/Graphene (DOPO/GN) ^e^	5	32	538	36.5	1.64	—	—	[166]
		119	294	114	—	—	—	[220]
organic phosphinate/Zinc borate (PFR/ZB) ^f^	30	116	209	123	1.27	—	—	[220]
	0	39	456	38	—	—	—	[167]
IFR contains melamine phosphate/cellulosic fibre containing polysilicic acid (IFR/Vis) ^g^	5	49	391	20.3	2.74	—	—	[167]
IFR contains melamine phosphate/cellulosic fibre containing polysilicic acid (IFR/Vis) ^g^	7.5	45	433	34	1.36	—	—	[167]
IFR contains melamine phosphate/cellulosic fibre containing polysilicic acid (IFR/Vis) ^g^	10	52	488	33.2	1.43	—	—	[167]
IFR contains melamine phosphate/cellulosic fibre containing polysilicic acid (IFR/Vis) ^g^	12.5	54	488	31.3	1.57	—	—	[167]
IFR contains melamine phosphate/cellulosic fibre containing polysilicic acid (IFR/Vis) ^g^	15	66	451	28.4	2.29	—	—	[167]
IFR contains melamine phosphate/cellulosic fibre containing polysilicic acid (IFR/Vis) ^g^	7.5	39	379	32.2	1.42	—	—	[167]
IFR contains melamine phosphate/cellulosic fibre containing polysilicic acid (IFR/Vis) ^g^	10	80	408	25.5	3.42	—	—	[167]
IFR contains melamine phosphate/cellulosic fibre containing polysilicic acid (IFR/Vis) ^g^	12.5	59	379	24.5	2.82	—	—	[167]
IFR contains melamine phosphate/cellulosic fibre containing polysilicic acid (IFR/Vis) ^g^	15	77	434	22.9	3.44	—	—	[167]
IFR contains melamine phosphate/cellulosic fibre containing polysilicic acid (IFR/Vis) ^g^	10	76	346	24.3	4.02	—	—	[167]
IFR contains melamine phosphate/cellulosic fibre containing polysilicic acid (IFR/Vis) ^g^	12.5	89	342	23	5.03	—	—	[167]
IFR contains melamine phosphate/cellulosic fibre containing polysilicic acid (IFR/Vis) ^g^	15	90	442	20.6	4.39	—	—	[167]
IFR contains melamine phosphate/cellulosic fibre containing polysilicic acid (IFR/Vis) ^g^	12.5	67	277	22.8	4.71	—	—	[167]
IFR contains melamine phosphate/cellulosic fibre containing polysilicic acid (IFR/Vis) ^g^	15	89	339	20.3	5.75	—	—	[167]
IFR contains melamine phosphate/cellulosic fibre containing polysilicic acid (IFR/Vis) ^g^	15	97	226	15.9	12.00	—	—	[167]
IFR contains melamine phosphate/cellulosic fibre containing polysilicic acid (IFR/Vis) ^g^	17.5	100	236	23.4	8.05	—	—	[167]
		125	857	50	—	—	—	[174]
Trisilanolisobutyl Polyhedral oligomeric silsesquioxane/triglycidyl isocyanurate (T8POSS/TGIC) ^h^	5	114	385	32	3.17	—	—	[174]
		40	525	62	—	—	—	[221]
IFR contains melamine phosphate/cellulosic fibre containing polysilicic acid (IFR/Vis) ^i^	5	24	365	67	0.80	—	—	[221]
IFR contains melamine phosphate/cellulosic fibre containing polysilicic acid (IFR/Vis) ^i^	7.5	31	290	41	2.12	—	—	[221]
IFR contains melamine phosphate/cellulosic fiber containing polysilicic acid (IFR/Vis) ^i^	10	28	242	36	2.62	—	—	[221]

^a^ Matrix: eight layers of woven E-glass fabric reinforced epoxy; ^b^ Matrix: eight layers of woven E-glass fabric reinforced epoxy; ^c^ Matrix: eight layers of woven E-glass reinforced film of multifunctional epoxy resin; ^d^ Matrix: eight plies of carbon fiber reinforced system HexFlow RTM6 (matrix) and HexForce G0939 (fabric); ^e^ Matrix: eight layers of woven roving glass fabric reinforced epoxy phenol novolak resin blend; ^f^ Matrix: epoxy fiber S2-glass panels; ^g^ Matrix: eight layers of woven E-glass reinforced epoxy; ^h^ Matrix: eight layers of woven glass Fiber Reinforced epoxy; ^i^ Matrix: eight ply woven roving E-glass fiber-reinforced epoxy.
